# A cryptic microdeletion del(12)(p11.21p11.23) within an unbalanced translocation t(7;12)(q21.13;q23.1) implicates new candidate loci for intellectual disability and Kallmann syndrome

**DOI:** 10.1038/s41598-023-40037-4

**Published:** 2023-08-10

**Authors:** Afif Ben-Mahmoud, Shotaro Kishikawa, Vijay Gupta, Natalia T. Leach, Yiping Shen, Oana Moldovan, Himanshu Goel, Bruce Hopper, Kara Ranguin, Nicolas Gruchy, Saskia M Maas, Yves Lacassie, Soo-Hyun Kim, Woo-Yang Kim, Bradley J. Quade, Cynthia C. Morton, Cheol-Hee Kim, Lawrence C. Layman, Hyung-Goo Kim

**Affiliations:** 1 Neurological Disorders Research Center, Qatar Biomedical Research Institute, Hamad Bin Khalifa University, Doha, Qatar; 2 Gene Engineering Division, RIKEN BioResource Research Center, Tsukuba, Japan; 3 Integrated Genetics, Laboratory Corporation of America Holdings, 3400 Computer Drive, Westborough, MA 01581 USA; 4 Division of Genetics and Genomics at Boston Children’s Hospital, Harvard Medical School, Boston, MA 02114 USA; 5 Medical Genetics Service, Pediatric Department, Hospital Santa Maria, Centro Hospitalar Universitário Lisboa Norte, Lisbon, Portugal; 6 Hunter Genetics, Waratah, NSW 2298 Australia; 7 University of Newcastle, Callaghan, NSW 2308 Australia; 8 Forster Genetics-Hunter New England Local Health District, Forster, NSW 2428 Australia; 9 Department of Genetics, Reference Center for Rare Diseases of Developmental anomalies and polymalformative syndrome, CHU de Caen Normandie, Caen, France; 10 Department of Human Genetics, Amsterdam University Medical Center, Amsterdam, the Netherlands; 11 Reproduction and Development Research Institute, University of Amsterdam, Amsterdam, the Netherlands; 12 Division of Genetics, Department of Pediatrics, Louisiana State University, New Orleans, LA 70118 USA; 13 Molecular and Clinical Sciences Research Institute, St. George’s, University of London, London, UK; 14 Department of Biological Sciences, Kent State University, Kent, OH 44242 USA; 15 Department of Pathology, Brigham and Women’s Hospital, Harvard Medical School, Boston, MA 02115 USA; 16 Departments of Obstetrics and Gynecology and of Pathology, Brigham and Women’s Hospital and Harvard Medical School, Boston, MA 02115 USA; 17 Broad Institute of MIT and Harvard, Cambridge, MA 02142 USA; 18 Manchester Centre for Audiology and Deafness, School of Health Sciences, University of Manchester, Manchester, UK; 19 Department of Biology, Chungnam National University, Daejeon, 34134 Korea; 20 Section of Reproductive Endocrinology, Infertility and Genetics, Department of Obstetrics and Gynecology, Augusta University, Augusta, GA USA; 21 Department of Neuroscience and Regenerative Medicine, Augusta University, Augusta, GA USA; 22 College of Health and Life Sciences, Hamad Bin Khalifa University, Doha, Qatar

**Keywords:** Genetics, Neuroscience, Diseases, Neurology, Pathogenesis

## Abstract

In a patient diagnosed with both Kallmann syndrome (KS) and intellectual disability (ID), who carried an apparently balanced translocation t(7;12)(q22;q24)*dn*, array comparative genomic hybridization (aCGH) disclosed a cryptic heterozygous 4.7 Mb deletion del(12)(p11.21p11.23), unrelated to the translocation breakpoint. This novel discovery prompted us to consider the possibility that the combination of KS and neurological disorder in this patient could be attributed to gene(s) within this specific deletion at 12p11.21-12p11.23, rather than disrupted or dysregulated genes at the translocation breakpoints. To further support this hypothesis, we expanded our study by screening five candidate genes at both breakpoints of the chromosomal translocation in a cohort of 48 KS patients. However, no mutations were found, thus reinforcing our supposition. In order to delve deeper into the characterization of the 12p11.21-12p11.23 region, we enlisted six additional patients with small copy number variations (CNVs) and analyzed eight individuals carrying small CNVs in this region from the DECIPHER database. Our investigation utilized a combination of complementary approaches. Firstly, we conducted a comprehensive phenotypic-genotypic comparison of reported CNV cases. Additionally, we reviewed knockout animal models that exhibit phenotypic similarities to human conditions. Moreover, we analyzed reported variants in candidate genes and explored their association with corresponding phenotypes. Lastly, we examined the interacting genes associated with these phenotypes to gain further insights. As a result, we identified a dozen candidate genes: *TSPAN11* as a potential KS candidate gene, *TM7SF3, STK38L, ARNTL2, ERGIC2, TMTC1, DENND5B*, and *ETFBKMT* as candidate genes for the neurodevelopmental disorder, and *INTS13, REP15, PPFIBP1*, and *FAR2* as candidate genes for KS with ID. Notably, the high-level expression pattern of these genes in relevant human tissues further supported their candidacy. Based on our findings, we propose that dosage alterations of these candidate genes may contribute to sexual and/or cognitive impairments observed in patients with KS and/or ID. However, the confirmation of their causal roles necessitates further identification of point mutations in these candidate genes through next-generation sequencing.

## Introduction

Kallmann syndrome (KS) is a clinically and genetically heterogeneous disorder characterized by the co-occurrence of idiopathic hypogonadotropic hypogonadism (IHH) and anosmia. IHH is primarily caused by a defective action of hypothalamic gonadotropin-releasing hormone (GnRH) through the hypothalamic-pituitary-gonadal axis, while anosmia is associated with dys/agenesis of the olfactory bulbs.

Several chromosomal rearrangements involving the 12q24 region have been reported in patients with KS and hypogonadism. These include balanced chromosomal translocations associated with KS t(7;12)(q22;q24)*dn*^1^, IHH t(4;12)(q25;q24.2)*dn*^2^, and severe primary hypogonadism t(1;12)(p32;q24)^3^, as well as a del(12)(q24.31q24.33) associated with IHH^4^. The molecular characterization of balanced chromosomal rearrangements linked to abnormal phenotypes has played a crucial role in positional cloning of disease genes^5–8^. For instance, the KS gene *WDR11* at 10q26.12 was identified through positional cloning of the balanced translocation t(10;12)(q26.12;q13.11)^9^. As there have been four reported chromosomal rearrangements^1–4^, suggesting the presence of a potential KS gene in the overlapping region of 12q24, we obtained the available lymphoblastoid cell lines from Patient 1, who carries a de novo apparently balanced translocation t(7;12)(q22;q24)^1^ from the Coriell Institute for Medical Research (www.coriell.org)^10^. Through positional cloning, we mapped and cloned both breakpoints, leading to the identification of a non-coding RNA, *RMST*, directly truncated at the chromosome 12 breakpoint^11^. This finding was confirmed by targeted breakpoint sequencing^8^ and genome sequencing^12^. We conducted further sequencing of five genes, including *RMST*, located at or in close proximity to the breakpoints on both chromosomes in a cohort of 48 recruited KS patients. However, no pathogenic variants were identified in these genes.

Although the translocation appeared balanced, microarray analysis revealed a previously unreported 4.7 Mb heterozygous microdeletion at 12p11.21-12p11.23 in Patient 1. This region likely contains candidate gene(s) for KS and ID. In silico genomic analysis of 15 copy number variations (CNVs) within this region identified one KS candidate gene, seven ID candidate genes, and four candidate genes for KS combined with ID.

These candidate genes will expedite the identification of pathogenic heterozygous variants by leveraging next-generation sequencing (NGS) databases. Specifically, they will aid in the search for such variants in individuals presenting with delayed/absent pubertal development, ID, or both. Additionally, given the phenotypic heterogeneity of mental disorders, these candidate genes will aid in the identification of disease-associated variants from databases of individuals with neurodevelopmental disorders (NDDs), including autism, thus expanding our understanding of the genetic basis of these conditions.

## Results

**Figure 1:**
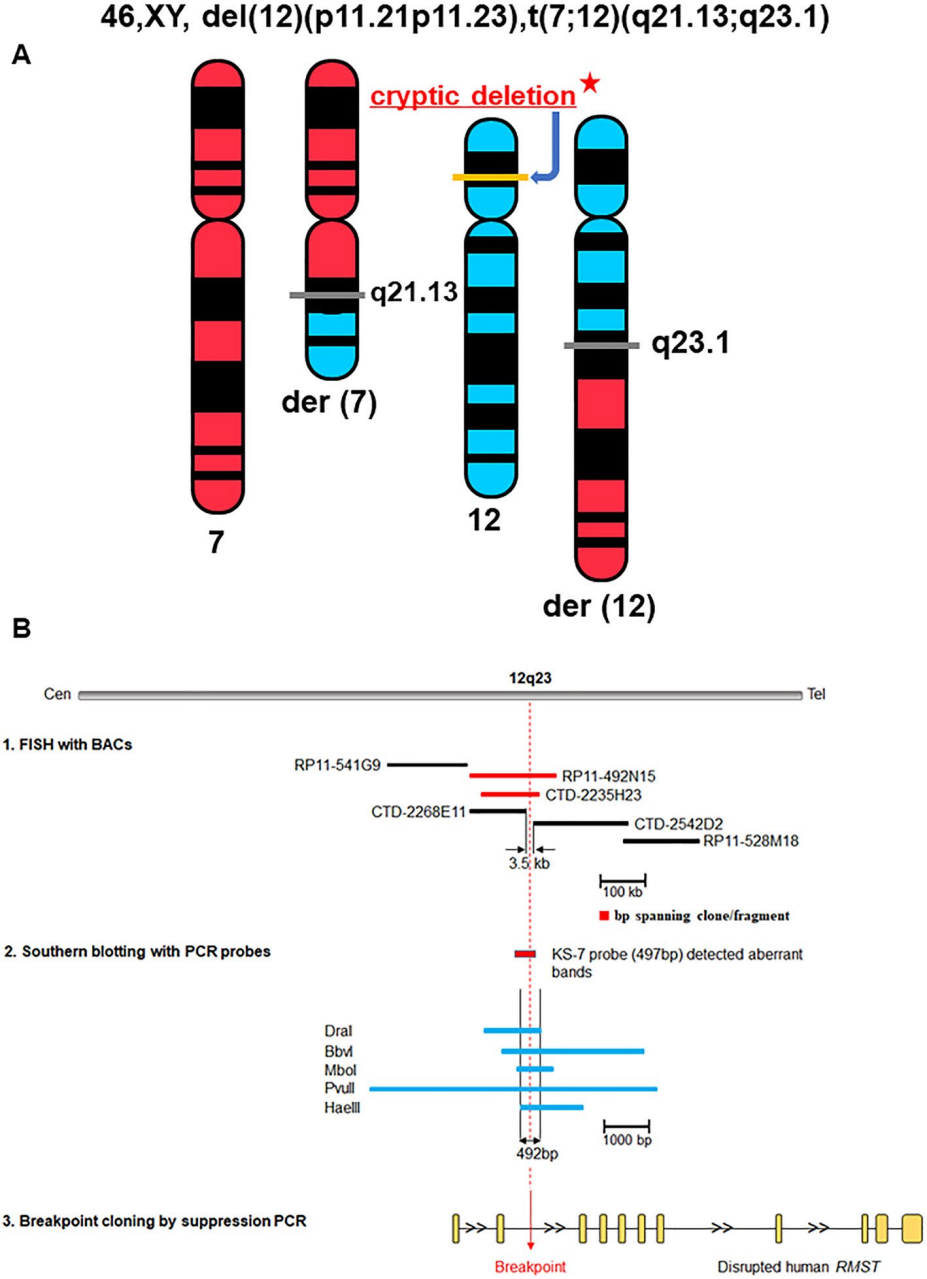
(**A**) Ideogram illustrating the revised t(7;12)(q21.13;q23.1)*dn* karyotype in patient 1, DGAP032. After breakage of two chromosomes, the reciprocal exchange of chromosome segments between chromosomes 7 and 12 has taken place, generating two derivative chromosomes in the patient with two horizontal gray bars at the breakpoint positions. On chromosome 12, the deleted cryptic segment at 12p11.21-12p11.23 identified was depicted as a horizontal yellow bar. (**B**) Physical mapping of the 12q23 translocation breakpoint of DGAP032 by FISH and Southern blot hybridization. Diagram shows breakpoint refined by FISH and Southern blot. For FISH, two BAC clones RP11-492N15 and CTD-2235H23 spanning the breakpoint, which is represented as a dashed vertical red line, were identified, and shown as red bars. The breakpoint was further narrowed to 3.5 kb between CTD-2268E11 and CTD-2542D2 shown as black bars. Southern blot analysis using Blot 4 with the probe KS-7 identified aberrant fragments of patient DNA digested with five different restriction enzymes (DraI, BbVI, MboI, PvuII, and HaeIII, Fig. 3B). The breakpoint was refined to 492 bp between the centromeric end of HaeIII and the telomeric end of DraI, which was then isolated with suppression PCR and sequenced. The breakpoint at 12q23.1 is located in intron 2 of *RMST* (NR_152618.1).

Positional cloning was employed to clone the translocation breakpoints and identify a gene potentially affected by the apparently balanced translocation in Patient 1, whose karyotype and clinical information were previously published (Fig. 1A)^1^. By constructing a bacterial artificial chromosome (BAC) contig and utilizing fluorescence in situ hybridization (FISH) and Southern blot analysis, we successfully mapped and narrowed down the translocation breakpoints. The cloning of the genomic breakpoint on chromosome 12 via suppression PCR revealed the truncation of the non-coding RNA *RMST* (Rhabdomyosarcoma 2 Associated Transcript, MIM 607045) at the 12q23.1 breakpoint (Fig. 1B). Additionally, the breakpoint on chromosome 7 was localized at 7q21.13, approximately 37 kb upstream of the predicted gene *ZNF804B* (Zinc Finger Protein 804B) with an unknown function. Consequently, the karyotype was revised to t(7;12)(q21.13;q23.1)*dn* (Fig. 1A)^11^. Although we initially considered *RMST* as a potential candidate gene due to other cases involving chromosome 12q24 with IHH during our initial positional cloning efforts between 2002 and 2008^11^, screening of five genes, including *RMST*, at and around both breakpoints in 48 KS patients did not reveal any pathogenic variants. Hence, the translocation breakpoint at 12q23 did not appear to harbor a positional candidate gene for KS.

**Figure 2:**
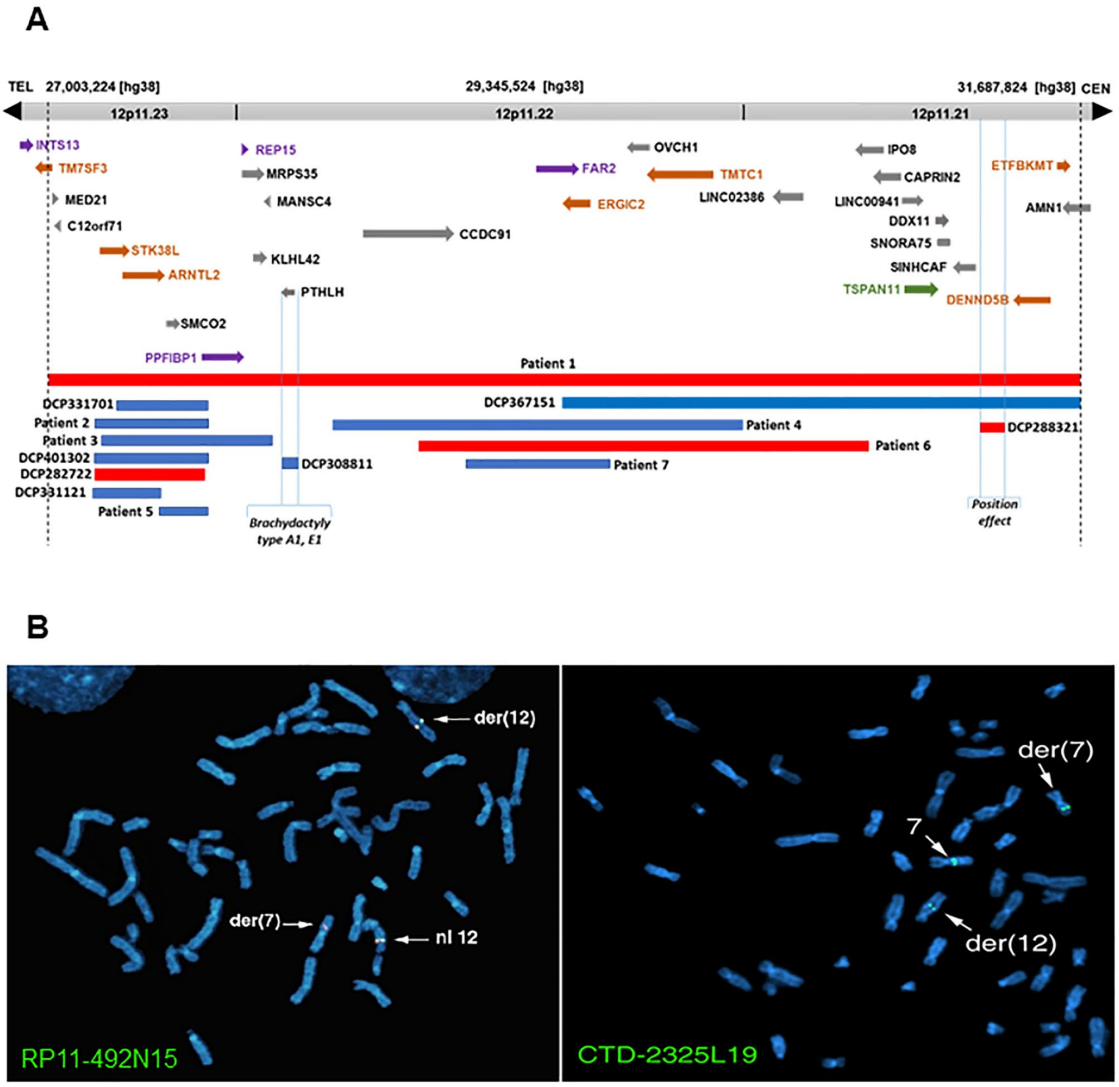
(**A**) Cryptic 4.7 Mb heterozygous deletion encompassing 29 genes located from 12p11.21 to 12p11.23. Eight heterozygous CNV cases from the DECIPHER database are denoted by DCP along with six heterozygous CNVs (patients 2-7) we recruited. These 14 CNVs are encompassed in Patient 1 to help narrow down the candidate gene region by in silico comparative genomic mapping. Red bars represent deletions, whereas blue bars represent duplications. One gene in green is a candidate for KS, whereas seven genes in brown are NDD candidates. Four genes in purple are chosen as candidates for KS combined with ID. Arrow indicates transcriptional direction of each gene. (**B**) FISH with two BAC clones spanning the breakpoints at 12q23.1 and 7q21.13, respectively. BAC clone RP11-492N15 shows normal signals on normal chromosome 12 and split signals on both derivative chromosomes 7 and 12. BAC clone CTD-2325L19 with normal signals on normal chromosome 7 and split signals on both derivative chromosomes 7 and 12.

Using aCGH with increased resolution, it is possible to detect CNVs (copy number variations) that may not be identified through karyotyping alone^13^. As cryptic deletions are frequently observed in seemingly balanced translocations^14,15^, we performed aCGH analysis, which uncovered a heterozygous 4.7 Mb deletion spanning 29 known and predicted genes at 12p11.21-12p11.23 (Fig. 2A). This cryptic heterozygous deletion in the patient with an apparently balanced translocation would not have been detected through previous chromosome analysis due to the limited resolution of karyotyping (approximately 5 Mb or higher)^16^. In order to identify positional candidate gene(s) for KS and/or ID, we recently recruited an additional six patients (Patients 2–7) with microdeletions and microduplications at 12p11.21-12p11.23 (Table 1 and Fig. 2A). Notably, these seven patients share common phenotypic features, including developmental delay (DD), ID, learning disability, and language/speech delay. Some patients also exhibit autism, craniofacial anomalies (CFA), and epilepsy (Table 1), suggesting the presence of neurodevelopmental gene loci within this region. Through *in-silico* comparative mapping of these seven patients, along with eight informative CNVs (https://decipher.sanger.ac.uk/, version 11.14)^17^ encompassing five microdeletions and ten microduplications within the microdeletion observed in Patient 1, we have implicated one KS candidate gene, seven neurodevelopmental candidate genes, and four candidate genes for KS combined with NDDs.

**Table 1:** Individual clinical features of seven patients with heterozygous deletions/duplications at 12p11.21-12p11.23 along with their demarcating genomic coordinates (hg38).

Subject ID	Patient 1: DGAP032	Patient 2: 50943	Patient 3: 31606	Patient 4: 022821	Patient 5: 295472	Patient 6: 370033	Patient 7: 293962
Genomic coordinates [hg38]	27,003,224–31,687,824	27,134,884–27,634,952	27,157,806–27,907,534	28,047,313–29,990,575	27,400,730–27,615,518	28,414,984–30,598,365	28,701,107–29,353,047
Cytogenetic band(s)	12p11.21-p11.23	12p11.23	12p11.22- p11.23	12p11.22	12p11.23	12p11.21-p11.22	12p11.22
Type of CNV	Deletion	Duplication	Duplication	Duplication	Duplication	Deletion	Duplication
CNV size	4.7 Mb	500 Kb	750 Kb	1.94 Mb	215 Kb	2.18 Mb	652 Kb
Inheritance	De novo with 46, XY, t(7;12)(q21;q23)*dn*	De novo	Unknown	De novo	Paternal	Paternal father & two paternal uncles have similar learning disabilities	Paternal father with mild learning problems with same syndactyly and tapering fingers
Method of detection	aCGH	aCGH	aCGH	aCGH	aCGH	aCGH	aCGH
Age	44 years	36 years	10 years	4 years	12 years 5 months	11 years	48 years
Sex	M	M	F	M	M	F	M
Ethnicity	Chippewa/French	Dutch	American	Argentinian	French	Portuguese	British
Developmental delay	_	+	+	+	+	+	+
Intellectual disability	+	+	N/A	+	+	+	+
Autism	_	+	_	+	+	_	_
Kallman syndrome	+	_	_	_	_	_	_
Cranial anomalies	+ sharp foramen	_	_	+ microcephaly	+ mild microcephaly	_	+ mild microcephaly
Facial dysmorphism	_	+	_	_	+	_	+
Learning disability	+	+	N/A	+	+	+	+
Epilepsy/seizures/spasms	_	_	_	_	N/A	_	_
Language/speech delay	N/A	+	+	+	+	+	+
Hearing loss	_	_	_	+	_	_	_
Hand/finger/feet/toe anomalies	+	_	_	+	+	+	+
Skeletal anomalies	+	_	_	+	_	_	_
Behavioral problems	_	+	_	_	+	+	_
Anxiety disorder	_	_	_	_	+	_	_
Hypotonia	_	_	N/A	+	_	_	_
Impaired motor skills	_	_	N/A	+	+	_	_
Dyslexia	_	+	N/A	+	_	_	+
ADHD	_	_	+	_	_	+	_

‘N/A’ denotes not available, while ‘-’ represents absence of the corresponding phenotype.

*ADHD* attention deficit hyperactivity disorder.

### Breakpoint region was refined to 3.5 kb at 12q23.1, and 87 kb at 7q21.13 by FISH mapping

FISH mapping was initiated using clones RP11-11O3 and RP11-77E2 on 7q21 and clones RP11-74K11 and RP11-1K22 on 12q23 to identify the breakpoints. These clones served as flanking markers for the breakpoints, and further experiments were conducted until the clones containing the breakpoints were identified, following the previously described methodology^7^. For the chromosome 12 breakpoint, final experiments utilized clones RP11-492N15 and CTD-2235H23, which hybridized to chromosome 12, der(12) chromosome, and der(7) chromosome, indicating that the translocation breakpoint of chromosome 12 resided within the sequence of these two BAC clones (Fig. 1B). The hybridization of SpectrumGreen labeled RP11-492N15 to chromosome 12 and both derivative chromosomes is illustrated in Fig. 2B. CTD-2542D2 was found to hybridize to chromosome 12 and der(7) chromosome, while CTD-2268E11 hybridized to chromosome 12 and der(12) chromosome, delineating the breakpoint region at 12q23 (Fig. 1B).

**Figure 3:**
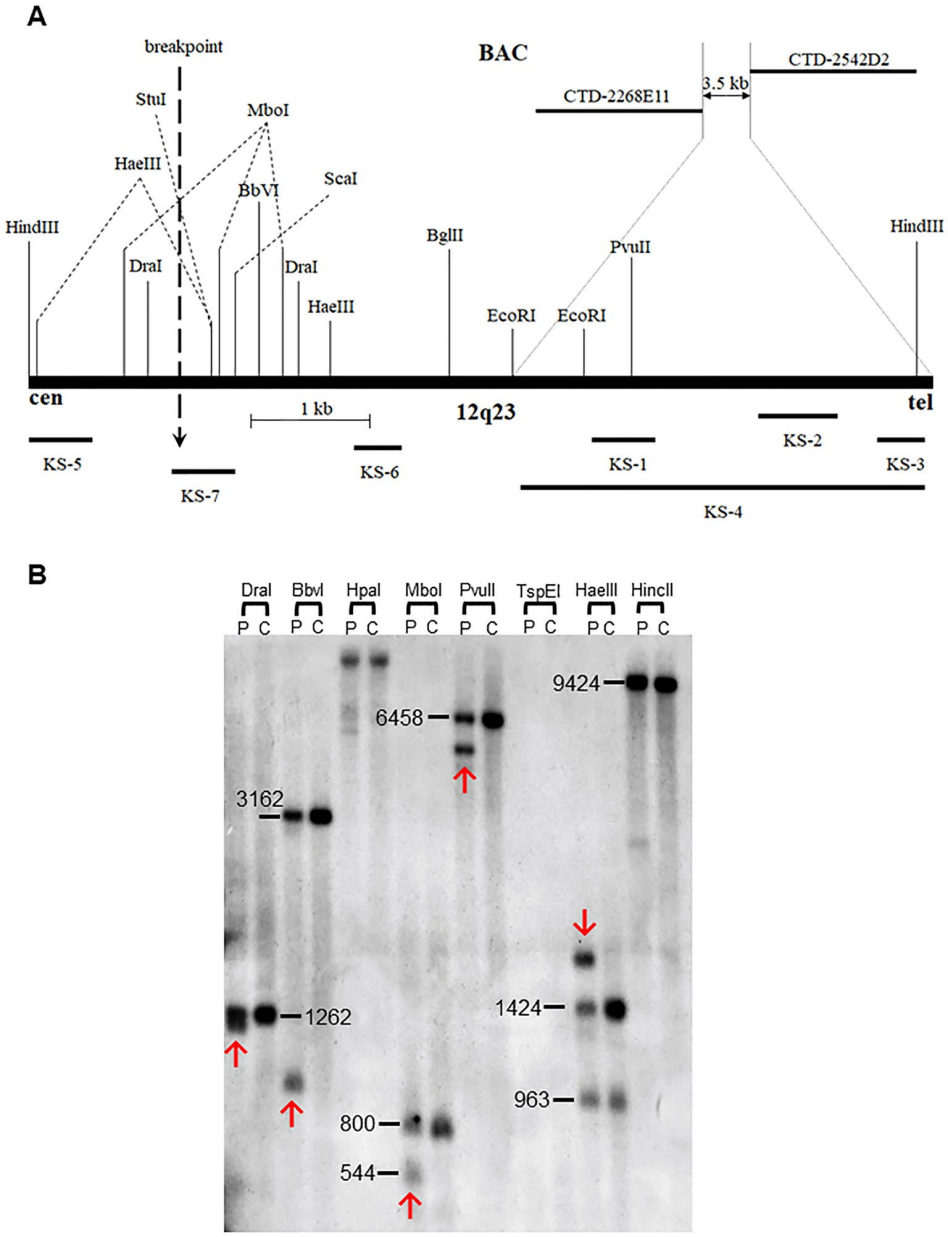
(**A**) Restriction map of a 7.3 kb HindIII genomic fragment (97,460,797–97,468,119/hg38) containing the breakpoint on 12q23. BamHI, HincII, HpaI and SnaBI do not have restriction sites on this map and only the relevant restriction sites in relation to the fragments detected by shown probes are indicated. Positions of the PCR-derived probes KS-1 to KS-7 used for the breakpoint mapping by Southern analysis are indicated below. Note that the breakpoint is located 2.7 kb upstream of the region narrowed by FISH. (**B**) Genomic DNA blots hybridized with probes from the 12q23 breakpoint region. Each lane contains genomic DNA digested with the designated restriction enzymes from either DGAP032 (P) or a normal control (C). Additional bands in the P lanes indicate novel restriction junction fragments generated by the interchromosomal exchange. The hybridization probe KS-7 detected aberrant bands indicated by red arrows containing breakpoints at 12q23. The numbers next to normal restriction fragment seen in both patient and control lanes indicate the size of genomic restriction fragment in bp.

These findings indicate that the chromosome 12 breakpoint was located within or adjacent to a 3.5 kb interval (chr12: 97,460,688–97,464,146 / hg38) between CTD-2542D2 and CTD-2268E11, based on the placement of end-sequenced BAC clones on the current genomic sequence map (Fig. 1B and Fig. 3A). This interval was positioned 874 bp upstream from the 5’ end of the *RMST* locus.

For the chromosome 7 breakpoint, mapping revealed its location within clone CTD-2325L19 (Fig. 2B), with clone RP11-46O13 being telomeric to the breakpoint. Based on the positions of these end-sequenced clones, the chromosome 7 breakpoint was determined to be within an 87 kb interval (data not shown). This interval did not contain any genes, and the nearest gene, ZNF804B, was approximately 11 kb beyond the telomeric boundary of the chromosome 7 breakpoint region defined by FISH mapping.

### Breakpoint region at 12q23.1 was refined to 492 bp by Southern blot analysis

To map the breakpoints where the disease gene in Patient 1 might be disrupted or dysregulated, Southern blot analysis was performed on the refined breakpoint region of chromosome 12 identified through FISH. Probes KS-1, KS-2, and KS-3 were sequentially hybridized to genomic DNAs from translocation patients and normal controls digested by BglII, DraI, EcoRI, EcoRV, HaeIII, HindIII, RsaI, and ScaI, using a filter (Blot 1). All three probes detected the same rearrangement observed in a 7.3 kb genomic HindIII fragment in the patient, which was absent in controls (data not shown). Thus, the breakpoint was located within a 7.3 kb HindIII restriction fragment (chr12: 97,460,797–97,468,119/hg38), which encompassed 3350 bp of the 3.5 kb putative breakpoint region narrowed down by FISH (Fig. 3A). To confirm that the aberrant band detected by HindIII was not a result of a polymorphism, probe KS-4 was hybridized to nylon membrane Blot 2 containing genomic DNAs digested by BamHI, StuI, HincII, NaeI, Sau3AI, SfoI, SnaBI, and SspI. An aberrant band was observed in the patient's genomic DNA lane with SnaBI, located below the 12.3 kb control SnaBI restriction fragment, which included the putative 7.3 kb breakpoint region narrowed by the HindIII restriction fragment. This confirmed that the aberrant band detected by HindIII was indeed caused by the chromosome rearrangement (data not shown).

As the putative breakpoint region defined by the HindIII rearranged fragment included a new 3.8 kb region, probes KS-5 and KS-6 were separately hybridized to genomic DNAs from the patient and normal control, digested by BamHI, StuI, HincII, DraI, EcoRI, HindIII, RsaI, and SspI, on two nylon membranes of Blot 3. Probe KS-5 detected rearranged fragments in the patient’s genomic DNAs digested with BamHI, StuI, HincII, EcoRI, and HindIII, while probe KS-6 detected rearranged fragments in the patient lanes with EcoRI and HindIII (data not shown). The breakpoint region was found within a 3.9 kb StuI restriction fragment (chr12: 97,466,625–97,470,522/hg38), overlapping with the breakpoint region of the 7.3 kb HindIII restriction fragment, further narrowing down the breakpoint to 1.5 kb (chr12: 97,466,625–97,468,119 / hg38).

To confirm and further refine the 1.5 kb breakpoint region, probe KS-7 was hybridized to a nylon membrane (Blot 4) containing digested genomic DNAs with DraI, BbvI, HpaI, MboI, PvuII, HaeIII, and HincII. Rearranged bands were detected in the patient DNA lanes with DraI, BbvI, MboI, PvuII, HaeIII, and HincII on the first membrane (Fig. 3B), narrowing down the breakpoint to a 492 bp region (chr12: 97,466,625–97,467,118/hg38, Fig. 1B).

### Breakpoint cloning of t(7;12)(q21.13;q23.1) identified *RMST* truncated at 12q23.1

#### Cloning of the breakpoint from derivative chromosome 12

**Figure 4:**
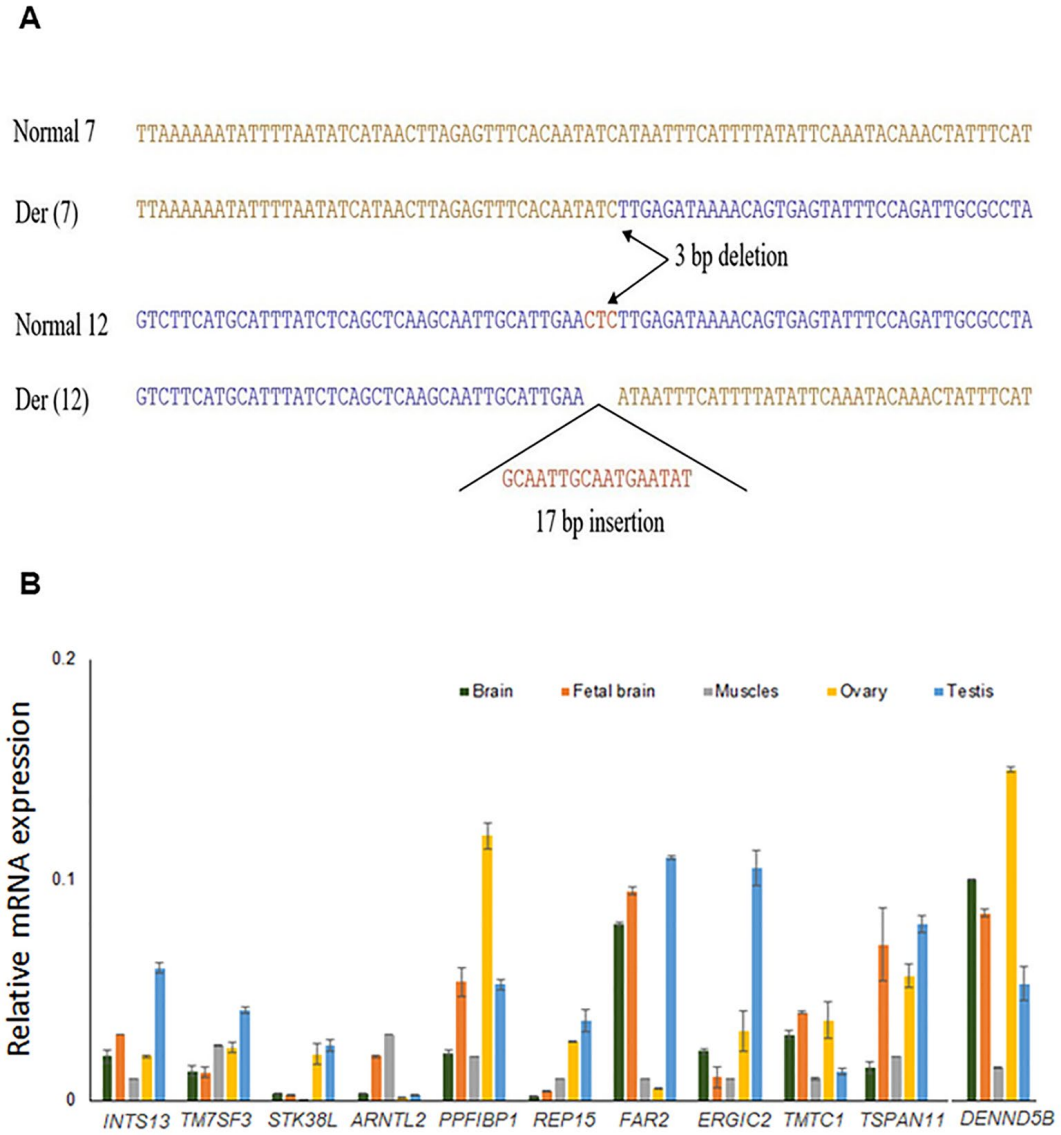
(**A**) Sequences of the junction fragment composed of two different chromosomes at the translocation breakpoints. Sequence comparison of the normal chromosomes 7 and 12 with der(7) and der(12) at the breakpoints junctions. Three bp sequence CTC from chr12 is deleted at the junction of der(7), and a 17 bp insertion was found at the junction of the der(12). (**B**) Transcript levels of *INTS13*, *TM7SF3*, *STK38L*, *ARNTL2*, *PPFIBP1*, *REP15*, *FAR2*, *ERGIC2*, *TMTC1*, *TSPAN11*, and *DENND5B* in five different human tissues (i.e. brain, fetal brain, muscle, ovary and testis) were determined by RT-qPCR. Varying levels of expression of these candidate genes were detected in different tissue samples.

The 3.5 kb junction fragment from derivative chromosome 12, which was detected on Blot 3 using probes KS-5 and KS-6 from EcoRI digestion, was amplified through two independent suppression PCRs^18^. The primers sets mentioned in Materials & Methods section were employed under the specific condition. The size of the resulting PCR products was 0.6 kb and 0.8 kb, respectively. Sequence analysis confirmed that these fragments corresponded to the junction fragments from the der(12). The genomic breakpoint was identified between positions 97,466,873 and 97,466,877 (hg38) at 12q23.1 (Fig. 4A), specifically within the second intron of *RMST* (Fig. 1B).

Since the junction fragment contained chromosome 7 sequences adjacent to the breakpoint, a BAC clone named CTD-2325L19 was identified through BLAT analysis on the Human Genome Browser (hg38). This clone mapped to 7q21 and was located within the 3.5 kb region narrowed down by FISH. BAC clone CTD-2325L19 also contained the chromosome 7 sequence of the junction fragment. FISH analysis (Fig. 2B, right picture) revealed hybridization signals of SpectrumGreen on chromosomes 7, der(7), and der(12), confirming the presence of the 7q21 breakpoint in the patient. Therefore, the results indicate that BAC clone CTD-2325L19 harbors the 7q21 breakpoint observed in the patient’s genome.

#### Cloning of the breakpoint from derivative chromosome 7

Since the sequence of the chromosome 7 region adjacent to the breakpoint of der(12) had been determined, a 2.3 kb junction fragment from der(7) was generated using nested PCR. This was achieved by employing primers located proximal to the 7q21.13 breakpoint and distal to the 12q23.1 breakpoint, as specified in the Materials & Methods section. Sequence analysis confirmed that this fragment corresponded to the junction fragment from der(7). The genomic breakpoint was identified between positions 88,722,752 and 88,722,753 (hg38) at 7q21.13 (Fig. 4A).

To investigate if there were any chromosomal alterations at the translocation site, two junction fragment sequences were examined. A comparison between the normal chromosome sequence at 7q21.13, the normal chromosome sequence at 12q23.1, and the sequences from the two junction fragments revealed an unknown 17 bp insertion (GCAATTGCAATGAATAT) in the der(12) junction fragment, as well as a 3 bp CTC deletion from chromosome 12 in the der(7) junction fragment^11^ (Fig. 4A).

### Identification of three candidate genes at 12q23.1 from t(7;12)(q21.13;q23.1)

To identify the gene associated with KS, we conducted mapping and sequencing of both translocation breakpoints. Previous studies reported two balanced translocations and one deletion 12q24 related to hypogonadism^2–4^. Therefore, we analyzed sequences upstream and downstream of the chromosome 12 breakpoint to search for a potential candidate gene. This analysis led to the identification of *RMST*. By comparing the genomic sequence, we determined that the translocation directly disrupted *RMST*, with the 12q23 breakpoint located within intron 2, downstream of the second exon of this gene (Fig. 1B)^11^. *RMST* is a long non-coding RNA specifically expressed in the developing brain.

Additionally, we found another candidate gene, *NEDD1* (Neural Precursor Cell Expressed, developmentally down-regulated, MIM 600372), mapped 513 kb upstream from the breakpoint. Furthermore, a pseudogene called *PAFAH1B2P2* (PAFAH1B2 pseudogene 2) was mapped 248 kb downstream from the breakpoint.

### Identification of two candidate genes at 7q21.13 from t(7;12)(q21.13;q23.1)

In de novo balanced translocation with an associated phenotype, the disease gene is often found near one of the two breakpoints^6,7,9,19^. We also examined the genes located at the chromosome 7 breakpoint. The nearest gene to the 7q21.13 breakpoint is *ZNF804B*, which is mapped 37 kb distal to the breakpoint. Another gene, *STEAP4* (Six-Transmembrane Epithelial Antigen of Prostate 4, also known as *STEAP4* Metalloreductase, MIM 611098), was mapped 416 kb proximal to the breakpoint^11^.

### Identification of the autosomal dominant candidate genes for KS and/or ID from del(12)(p11.21p11.23)

aCGH analysis revealed the presence of a heterozygous 4.7 Mb interstitial microdeletion spanning the chromosomal band at 12p11.21-12p11.23 (Chr12: 27,003,224–31,687,824/hg 38) (Figs. 1A and 2A). Consequently, based on the abnormal karyotype and aCGH findings in Patient 1, the revised nomenclature is 46,XY,t(7;12)(q21.13;q23.1)*dn*, arr[hg 38] 12p11.23p11.21(27,003,224–31,687,824)x1 (Fig. 1A), and this information was reported back to Coriell.

Through in silico comparative genomic mapping^20–24^ at 12p11.21-12p11.23, we identified a total of 12 autosomal dominant positional candidate genes associated with KS, ID, or both. Among them, *TSPAN11* is a candidate gene specifically related to KS, while *TM7SF3, STK38L, ARNTL2, ERGIC2, TMTC1, DENND5B*, and *ETFBKMT* are candidate genes associated with ID. Additionally, the four candidate genes potentially linked to both KS and ID are *INTS13, PPFIBP1, REP15,* and *FAR2* (Fig. 2A and Table 2).

**Table 2:** Twelve autosomal dominant positional candidate genes identified by in silico CNV mapping at 12p11.21-12p11.23 from telomeric to centromeric direction.

Gene (MIM #)	Candidate gene for	Chr. location	Function	Variants reported in NDD patients, interacting NDD genes, and animal KO phenotype
*INTS13* (aka *ASUN*) (615079)	KS+NDD	12p11.23	Involved in regulation of mitotic cell cycle	Two nonsense^45,133^, one synonymous^27^, and one missense^134^ variants in NDD patients Critical regulator of spermatogenesis in Drosophila^98^ Germline expression of mouse Asun rescued sterility and dynein mislocalization in Asun mutant flies^99^
*TM7SF3* (605181)	NDD	12p11.23	Involved in the inhibition of cytokine-induced death of pancreatic beta cells	Five missense^27,38,134,135^ variants in NDD patients TM7SF3 is interacting with HNRNPL^39^, a gene involved in ID^40^
*STK38L* (615836)	NDD	12p11.23	Involved in the regulation of structural processes in differentiating and mature neuronal cells	Two missense^46,134^, one synonymous^134,135^ and one nonsense^45^ variants in NDD patients arbor-specific changes in dendritic complexity seen in the hippocampus of Stk38l KO mice^41^
*ARNTL2* (2614517)	NDD	12p11.23	Transcriptional activator, which forms a core component of the circadian clock	One synonymous^135^, two missense^47,134^, and one nonsense^48^ variants in NDD patients ARNTL2 is interacting with CTTNBP2^49^*,* UBE3A^51^*,* and PER2^55^*,* three genes involved in NDDs
*PPFIBP1* (603141)	KS+NDD	12p11.22-p11.23	May regulate the disassembly of focal adhesions	Four nonsense^136^, two missense^134–136^, one splice^136^, and three frameshift^136,137^ variants in NDD patients PPFIBP1 is interacting with TACR3^55^, a gene involved in HH with or without anosmia^100^ PPFIBP1 is interacting with YWHAG^55^, KRAS^60^, NRAS^101^, HRAS^101^, CUL3^102^, and SNAP29^55^, six genes involved in NDDs
*REP15* (610848)	KS+NDD	12p11.22	Regulates transferrin receptor recycling from the endocytic recycling compartment	*Rep15* KO Mice have an abnormal behavior phenotype (http://www.informatics.jax.org/marker/MGI:1913782) REP15 is interacting with TLK2^55^, a gene involved in NDD^110^, ASD^58^, ID^138^, and Schizophrenia^111^ REP15 is interacting with SLC4A2^55^. Slc4a2 KO Mice revealed an interruption in spermiogenesis leading to infertility^109^
*FAR2* (616156)	KS+NDD	12p11.22	Catalyzes the reduction of saturated but not unsaturated C16 or C18 fatty acyl-CoA to fatty alcohols	One missense^134,135^ variant in unrelated NDD patients FAR2 interacts with PEX19^38,59,91^, a gene involved in NDDs^27,38^ FAR2 is interacting with ZP2^55^, a gene related to oocyte maturation defect leading to female infertility^113^. Homozygous Zp2 -/- mouse females were sterile^114^ FAR2 is interacting with GRPR^59^, a gene disrupted at the breakpoint in a patient^46^, XX, t(X;8)(p22.13;q22.1) with autism and multiple exostoses^139^ FAR2 is interacting with GRPR^29^. GRPR deficient mice exhibits decreased inhibition of principal neurons, enhanced long-term potentiation (LTP), and greater and more persistent long-term fear memory^140^
*ERGIC2* (612236)	NDD	12p11.22	Possible role in transport between endoplasmic reticulum and Golgi	One missense^134^, and one frameshift^58^ variants in NDD patients ERGIC2 is interacting with SLC39A8^55,59^*,* CUX1^60^*,* RAB3GAP1^60^, and RAB3GAP2^60^*,* four genes involved in NDDs
*TMTC1* (615855)	NDD	12p11.22	Transfers mannosyl residues to the hydroxyl group of serine or threonine residues	One missense^27,38^, one synonymous^134^, and one splice^134^ variants in NDD patients TMTC1 is interacting with BCOR^72^*,* and VIRMA^73^*,* two genes involved in NDDs
*TSPAN11* tetraspanin 11	KS	12p11.21	Integral membrane protein, regulating cell adhesion, motility, and synapse formation, interacts with integrins	One missense^37^ variant in KS patient
*DENND5B* (617279)	NDD	12p11.21	Guanine nucleotide exchange factor (GEF) which may activate RAB39A and/or RAB39B	One synonymous^135^, and one missense^26,27,45,117^ variants in NDD patients DENND5B is interacting with RAB11A^60^, and GRB10^84^*,* two genes involved in NDDs
*ETFBKMT* (615256)	NDD	12p11.21	Enables heat shock protein binding activity and protein-lysine N-methyltransferase activity	One missense^134,135^ and one synonymous^27,134,135^ variants in NDD patients ETFBKMT is interacting with TUBB2A^90^, TUBB4A^90^, DARS2^55^, and GLS^91^*,* four genes involved in NDDs

They include one gene for KS, seven genes for NDDs, and four genes for KS coupled with NDDs.

*NDD* denotes neurodevelopmental disorder, *ID* intellectual disability, *HH* hypogonadtropic hypogonadism, *ASD* autism spectrum disorder, *KS* Kallmann syndrome.

### Validation of putative candidate genes using tissue-specific RT-qPCR

To assess the functional significance of 11 out of 12 positional candidate genes in tissues relevant to the phenotype, we conducted RT-qPCR experiments to measure transcript levels in five distinct human tissues: brain, fetal brain, muscles, ovary, and testis. It is important to note that gene expression patterns can vary due to spatiotemporal regulation, as well as other factors such as the RNA isolation process and detection techniques employed. To establish a reference for the expression of our genes of interest, we utilized commercially available human RNA samples. This approach was necessary due to the diverse expression patterns observed in publicly available resources like the GTEx Portal (https://gtexportal.org/home/) and NCBI (https://www.ncbi.nlm.nih.gov/).

Among our candidate genes, *TSPAN11*, implicated in KS, exhibited high expression levels in testis and ovary. Additionally, five out of seven NDD-candidate genes (*TM7SF3, ARNTL2, ERGIC2, TMTC1*, and *DENND5B*) displayed good expression levels in brain and fetal brain tissues. Our four candidate genes for KS+NDD (*INTS13, PPFIBP1, REP15,* and *FAR2*) demonstrated expression in ovary, testis, and both adult and fetal brain tissues (Fig. 4B). The expression of these genes in relevant disease-associated organs suggests their potential involvement in the clinical phenotype when mutated. *ETFBKMT*, identified as an additional ID candidate gene upon re-evaluation of all 29 genes at 12p11.21-12p11.23, was not included in this experiment.

## Discussion

To date, there have been reports of three balanced translocations and one microdeletion involving 12q24 associated with hypogonadism or KS^1–4^. In a case reported in 1983, all three brothers of Vietnamese Chinese origin were found to have severe primary hypogonadism with a karyotype of 46,XY,t(1;12)(p32;q24)^3^. Another case, published in 1990, described an apparently balanced translocation t(7;12)(q22;q24) in a male with KS and ID^1^. In 1994, a Turkish male patient exhibited isolated hypogonadotropic hypogonadism (IHH) and a lack of secondary sexual characteristics, but with normal olfaction, due to a balanced chromosomal translocation between the distal q arms of chromosomes 4 and 12, t(4;12)(q25;q24.2)^2^. Following the publication of these three cases involving hypogonadism, a de novo interstitial deletion del(12)(q24.3q24.33) was described in a male individual with ambiguous genitalia and DD in 1999^4^.

We postulated that the four previously reported chromosomal rearrangements affecting the 12q24 region could be attributed to the haploinsufficiency of a specific gene responsible for KS or isolated hypogonadotropic hypogonadism (IHH). Successful positional cloning of KS-related genes at the translocation breakpoints has been demonstrated in previous studies^9,19^. To explore the potential contribution of the translocation to the phenotypes observed in male Patient 1 with t(7;12)(q22;q24)^1^, we conducted mapping and sequencing of both translocation breakpoints with the aim of identifying genes that might be involved in KS, ID, or both^11^.

The translocation breakpoint was precisely mapped through physical mapping using FISH and Southern blot hybridization, leading to the identification of five genes at both breakpoints. The gene *ZNF804B*, located 37 kb distal to the 7q21.13 breakpoint, was found to be the closest gene to this breakpoint, while *STEAP4* mapped 416 kb proximal to it. At the 12q23.1 breakpoint, the non-coding RNA *RMST* was directly disrupted, with *NEDD1* and *PAFAH1B2P2* mapping closest to the proximal and distal sides of the breakpoint, respectively. Based on the molecular analysis results, the cytogenetic band locations on both chromosomes 7 and 12 of the apparently balanced chromosome translocation were accurately revised as t(7;12)(q21.13;q23.1)^8,11,12^. Considering the overlapping phenotypes of KS observed in previously reported chromosomal rearrangements, we hypothesized that the causative gene for KS is located on chromosome 12. We screened three selected candidate genes (*RMST, NEDD1, PAFAH1B2P2*) for mutations based on their proximity to the 12q23.1 breakpoint. In intron 2, *RMST* was found to be disrupted, and mutation screening of this gene was performed in 48 KS patients who tested negative for *ANOS1* and *FGFR1*. In a KS male patient, we identified a heterozygous nucleotide change (C/C to C/T at the 214th nucleotide in exon 10 of *RMST* NR_152618.1), which was later determined to be a polymorphism as the patient's two healthy sisters shared the same nucleotide change. Interestingly, the patient and his mother with anosmia were found to have an *FGFR1* mutation (c.821G>A, p.E274G)^25^*,* suggesting *FGFR1* as the cause of the phenotype in this family. We also screened *PAFAH1B2P2* and *NEDD1*, which map 248 kb downstream and 513 kb upstream from the breakpoint, respectively, in the same cohort of 48 recruited KS patients, but no evidence of mutations was found.

Within the breakpoint region at 7q21.13, two additional genes were mapped. *ZNF804B*, located 37 kb downstream from the breakpoint, is the closest gene to the breakpoint^8^. ZNF804B belongs to the zinc finger protein family and consists of four exons. Although it has not been extensively studied, previous research suggests its implication in autism spectrum disorder (ASD) and NDDs^26,27^. Therefore, dysregulation of ZNF804B due to position effect may contribute to ID observed in Patient 1. On the other hand, *STEAP4*, located at 7q21.12, is situated 416 kb upstream from the breakpoint. Acting as a metalloreductase, STEAP4 plays a role in adipocyte development and metabolism, primarily within the Golgi apparatus. Mutation screening of both *ZNF804B* and *STEAP4* in the cohort of 48 recruited KS patients did not reveal any potential disease-causing mutations; however, polymorphisms were detected. Furthermore, we also screened for mutations in *ANOS1* and *FGFR1* in the patient with the t(7;12) translocation but did not find any potential disease-causing mutations (data not shown). It is important to note that the possibility of mutations in other causative genes for KS in this patient cannot be excluded.

Approximately 6% of carriers of balanced translocations exhibit abnormal phenotypes^28^ due to disruption of genes at the breakpoints or dysregulation (position effect), resulting in reduced gene expression caused by separation from their cis regulatory elements^29^. However, it is worth mentioning that 40% of patients with apparently balanced translocations have been reported to carry at least one deletion at one of the breakpoints or in other genomic regions, indicating that deletions may be common in seemingly balanced chromosome rearrangements^14^.

After not finding any mutations in the five candidate KS genes at or near the breakpoints of the apparent balanced translocation, we conducted aCGH analysis, which revealed a significant finding. We identified a cryptic 4.7 Mb submicroscopic microdeletion at 12p11.21-12p11.23. Interestingly, this cryptic microdeletion was not previously detected in molecular-level studies of the breakpoints in Patient 1 (DGAP032)^8,11,12^.

While there have been reports of chromosomal variations, including copy number variations (CNVs), in lymphoblastoid cell lines (LCLs), it is worth noting that these studies did not associate chromosome 12 with significantly different numbers of CNVs^30^. Therefore, it is unlikely that del(12)(p11.21p11.23) is solely a culture-induced artifact, although we cannot completely rule out this possibility. Unfortunately, due to the unavailability of parental samples, we were unable to determine the de novo status of this microdeletion.

In cases where a patient has concomitant genomic rearrangements, such as an unbalanced translocation and a simultaneous translocation-unrelated microdeletion or microduplication, it has been observed that the disease gene can be located at one of the translocation genomic breakpoints, although this is rare. For instance, in a female patient affected with autism and ID with t(14;21)(q21.1;p11.2)*dn* and 2.6 Mb of microdeletion comprising 15 genes at 2q31.1, the causative gene *LRFN5* (Leucine-Rich Repeat and Fibronectin Type III Domain-Containing Protein 5, MIM 612811) was found dysregulated at the 14q21.1 translocation breakpoint^31^. However, in most cases, the disease gene is within a copy number variant (CNV), as demonstrated by the identification of two positional ID candidate genes, *VAMP8* (Vesicle-Associated Membrane Protein 8, MIM 603177) and *RNF181* (Ring Finger Protein 181, MIM 612490) at 2p11.2, in a patient with an unbalanced t(8;10)(p23.3;q23.2) involving a cryptic 390 kb duplication region at 2p11.2^20^.

The result of aCGH analysis in Patient1 revealed a heterozygous 4.7 Mb interstitial deletion genes at 12p11.21-12p11.23 (chr12: 27,003,224–31,687,824/hg 38), spanning 29 genes (Figs. 1A and 2A). This deletion, which differs from the genomic breakpoints of the reciprocal translocation, is potentially involved in KS, ID, or both. Supporting this hypothesis is DECIPHER case 284660 (not listed in Fig. 2A), carrying a 7.09 Mb heterozygous deletion (chr12:22,444,774-29,533,886 [hg38]) and exhibiting cryptorchidism and mild global DD (https://decipher.sanger.ac.uk/). This microdeletion overlaps with a 2.53 Mb genomic region in our patient with KS (chr12:27,003,224–29,533,886 [hg38]).

In addition to KS, Patient 1, with an unbalanced chromosome translocation, also presented with ID^1^. While recent reports have identified genes associated with IHH and ID^32,33^, multiple genes contributing to the comprehensive phenotype are plausible explanations, as seen in contiguous gene deletion syndromes like Potocki-Shaffer-Syndrome^6^ or a deletion on chromosome X causing KS coupled with ID^34^.

Given the shared neurodevelopmental phenotypes observed in our seven CNV patients, as well as additional eight unpublished CNV cases from the DECIPHER database, this microdeletion, encompassing 29 genes, is likely to harbor the disease genes involved in these common phenotypes (Fig. 2A, Tables 1 and 3).

**Table 3:** Summary of the eight heterozygous DECIPHER CNV cases at 12p11.21-12p11.23.

Decipher ID	Copy number variation	Cytogenetic band	Genomic coordinates [hg38] (size)	Inheritance	Phenotype
DCP401302	Duplication	12p11.23	27,134,884–27,634,952 (500 kb)	De novo	*Arrhythmia, autistic behavior, hypertelorism, intellectual disability, mitral regurgitation, thick lower lip vermilion*
DCP282722	Deletion	12p11.23	27,153,357–27,607,134 (454 Kb)	De novo	*Cleft palate, microcephaly, secundum atrial septal defect,* small weight between 0.4th and 2nd centiles
DCP331121	Duplication	12p11.23	27,153,357–27,449,723 (296 kb)	Maternal	*Seizures,* epilepsy, prepubertal
DCP331701	Duplication	12p11.23	27,178,339–27,607,134 (429 kb)	Maternal	*Dystonia,* mild developmental delay, no dysmorphism, prepubertal
DCP308811	Duplication	12p11.22	27,929,322–27,999,621 (70 Kb)	Maternal	*Radial bowing, short humerus, brachydactyly type A1*
DCP367151	Duplication	12p11.21-12p11.23	29,151,249–31,658,390 (2.51 Mb)	Unknown	*Autistic behavior, behavioral abnormality, intellectual disability, macrocephaly, poor fine motor coordination*
DCP288575	Deletion	12p11.22	29,755,672–29,930,757 (175 Kb)	Paternal	*Autistic behavior,* normal intelligence, autism spectrum disorder, prepubertal
DCP288321	Deletion	12p11.21	31,128,897–31,240,772 (112 Kb)	Unknown	*Dystonia, Charcot-Marie-Tooth type 1A, prepubertal*

Out of the 15 heterozygous CNVs at 12p11.2, ten cases were duplications, while the remaining five cases were deletions (Fig. 2A, Tables 1 and 3). Each case exhibited at least one neurodevelopmental phenotype. In some instances, the CNVs were inherited from one parent with an unknown phenotype, while the inheritance status of the others remains unknown (Tables 1 and 3). By examining the pHaplo/pTriplo scores, we evaluated the potential haploinsufficiency and triplosensitivity of our 12 candidate genes using a dosage-sensitivity metrics catalog encompassing data for 18,641 genes^35^. Among the analyzed genes, seven demonstrated concurrent haploinsufficiency and triplosensitivity, as indicated by the following scores: INTS13 (ASUN): 0.97/0.82, TM7SF3: 0.52/0.40, STK38L: 0.77/0.94, PPFIBP1: 0.81/0.66, TMTC1: 0.50/0.50, TSPAN11: 0.31/0.90, DENND5B: 0.96/0.96. This suggests that duplications and deletions may be used to further refine the candidate gene region at 12p12.11-12p11.23. This observation is supported by the phenotypic similarities observed in patients with both deletions and duplications in this region. Any copy number variation (CNV) within this region has the potential to disrupt the precise stoichiometric control of gene expression at the protein level, thereby contributing to phenotypic alterations^36^.

Based on in silico comparative genomic analysis at 12p11.21-12p11.23, we propose *TSPAN11* (Tetraspanin 11) as a putative candidate gene for KS. A missense variant c.203G>A (NM_001080509.3) in *TSPAN11* was identified in a KS patient, resulting in an amino acid substitution from Glycine to Aspartic acid at position 68^37^. This variant shows high deleterious CADD score of 25.8 (HG38).

Furthermore, we have identified seven candidate genes for ID or NDD: *TM7SF3*, *STK38L*, *ARNTL2*, *ERGIC2*, *TMTC1*, *DENND5B* and *ETFBKMT* (Table 2). Candidate genes for ID and NDDs were identified by searching multiple human disease databases, such as HGMD, MGI, BioGrid, and STRING. Selection criteria included reported nucleotide variants associated with NDDs in the candidate genes or their interacting genes. We also assessed their interactions with known NDD genes and considered the behavioral phenotypes observed in knockout mice.

The putative position effect of *TM7SF3,* along with the inclusion of *STK38L* and *ARNTL2* in CNVs at 12p11.23, in addition to their sporadic variants reported in NDD patients, is likely to provide an explain their candidacy. Specifically, a patient with NDD was found to have a de novo missense variant in *TM7SF3* (Transmembrane 7 Superfamily Member 3, MIM 605181)^27,38^. Furthermore, an interacting protein of *TM7SF3*^39^, *HNRNPL* (Heterogeneous Nuclear Riboprotein L, MIM 603083), was described to have one missense variant in an ID patient^40^.

In mouse hippocampal neurons^41^, *STK38L* (Serine/Threonine Kinase 38 Like, aka *NDR2*, Nuclear Dbf2 Related Kinase 2, MIM 615836) regulates the morphology and division of neuronal cells^42–44^, as well as integrin-dependent dendritic and axonal growth. Consequently, Stk38l KO mice exhibit arbor-specific alterations of dendritic complexity in the hippocampus^41^. De novo nonsense and missense variants in *STK38L* have been identified in individuals with ASD^45^ and schizophrenia^46^, respectively. Genes mutated in schizophrenia are also mutated in autism and ID^46^.

The third candidate gene of ID at 12p11.23 is *ARNTL2* (Aryl Hydrocarbon Receptor Nuclear Translocator-Like Protein 2, MIM 614517), and its missense and nonsense variants were reported in patients with autism^47^, as well as in those with developmental and epileptic encephalopathy^48^. Proteins that physically interact with one another frequently participate in the same biological activity, and mutations in these genes may result in similar clinical features. Among the interactors of ARNTL2, *CTTNBP2* (Cortactin Binding Protein 2, MIM 609772)^49^ stands out, as it has been associated with 26 de novo genetic variants in probands with autism/DD^50^. Furthermore, another noteworthy interactor *UBE3A* (Ubiquitin-Protein Ligase E3A, MIM 601623)^51^, a well-known Angelman syndrome gene^52^, has two frameshift variants reported in autistic individuals^53,54^. Another interactor^55^ *PER2* (Period Circadian Regulator 2, MIM 603426), has been described with eight variants in individuals with autism^45,56,57^.

At 12p11.22, two ID candidate genes, *ERGIC2* and *TMTC1*, have been identified. A frameshift variant in *ERGIC2* (Endoplasmic Reticulum-Golgi Intermediate Compartment Protein 2, MIM 612236) has been reported in an individual with ASD^58^. ERGIC2 physically interacts with SLC39A8 (Solute Carrier Family 39, Member 8, MIM 608732)^55,59^ and CUX1 (Cut-Like Homeobox, 116896)^60^, which are associated with autosomal recessive syndromic ID^61^ and non-syndromic ID/DD^62^, respectively. ERGIC2 also interacts with two catalytic subunits of Rab GTPase activating proteins RAB3GAP1 (RAB3 GTPase-Activating Protein, Catalytic Subunit, 602536)^60^ and RAB3GAP2 (RAB3 GTPase-Activating Protein, Noncatalytic Subunit, MIM 609275)^60^. Homozygous mutations in these genes cause autosomal recessive Warburg Micro syndrome, characterized by developmental abnormality of the central nervous system^63–65^.

On the other hand, a de novo missense variant in *TMTC1* (TransMembrane and Tetratricopeptide repeat Containing 1, MIM 615855) was found in a child with NDD^27^. The Tetratricopeptide repeat (TPR) structural motif present in TMTC1 is also found in other genes such as *NAA15*^66^, *OGT*^67–69^, *TANC2*^70^, and *TTC25*^71^, all of which are associated with autism and ID. TMTC1 interacts with BCOR (BCL6 Corepressor, 300485)^72^ and VIRMA (Vir like M6A Methyltransferase Associated, MIM 616447) (aka KIAA1429)^73^. The mutations of the former cause Lenz microphthalmia, an X-linked syndromic ID^74^, whereas the variants of the latter have been found in individuals with ASD^45^, DD^27^, schizophrenia^45,46^, and Tourette syndrome^75^.

At 12p11.21, we have identified two additional ID candidate genes. One of these genes is *DENND5B* (DENN Domain Containing 5B, MIM 617279), a guanine nucleotide exchange factor (GEF) responsible for activating small GTPases, which function as molecular switches in intracellular signaling pathways. Several GEFs have been associated with NDDs, including *IQSEC2* associated with X-linked ID, and variants in *HERC1*^26,76^, *TRIO*^77,78^, *ARHGEF9*^79,80^, and *ARHGEF10*^81^, reported in patients with ID, epilepsy, and/or autism. Notably, *VAV3* (VAV Guanine Nucleotide Exchange Factor 3, MIM 605541) identified as an NDD candidate gene at 1p13.3, based on its KO mouse phenotype, genomic position, and reported variants, also functions as a GEF^82^. Additionally, DENND5B interacts with RAB11A (RAS-Associated Protein, MIM 605570)^83^ and GRB10 (Growth Factor Receptor-Bound Protein 10, MIM 601523)^84^, and variants in these interacting genes have been found in individuals with developmental and epileptic encephalopathies^48,85^.

The second ID candidate gene at 12p11.21 is *ETFBKMT* (electron transfer flavoprotein subunit beta lysine methyltransferase, MIM 615256), also known as *METTL20* (Methyltransferase like 20), which acts as a lysine methyltransferase. Other lysine methyltransferases such as *KMT2C* (Lysine Methyltransferase 2C, MIM 606833, aka MLL3), *SETD1B* (SET Domain Containing 1B, MIM 611055, aka KMT2G, Lysine-specific Methyltransferase 2G)^22,86^, *EHMT1* (Euchromatin Histone Lysine Methyltransferase 1, MIM 607001)^87,88^, and *KMT5B* (Lysine Methyltransferase 5B, MIM 610881)^89^ are well known to be associated with NDDs. On the protein level, ETFBKMT interacts with TUBB2A (Tubulin, Beta-2A, MIM 615101)^90^, TUBB4A (Tubulin, Beta-4A, MIM 602662)^90^, DARS2 (Aspartyl-tRNA Synthetase 2, MIM 610956)^55^, and GLS (Glutaminase, MIM 138280)^91^, all of which are associated with NDDs. *TUBB2A* is associated with seizures, ID and DD^92^, while *TUBB4A* mutations cause leukoencephalopathy hypomyelination with atrophy of the basal ganglia and cerebellum^93^. *DARS2* is genetically linked to leukoencephalopathy with brain stem and spinal cord involvement^94,95^. Trinucleotide expansion in *GLS* causes DD, ataxia, and cerebellar atrophy^96^.

*DDX11* (DEAD/H-Box Helicase 11, MIM 601150) located at 12p11.21, is associated with autosomal recessive Warsaw Breakage syndrome, which includes ID^97^. However, it was excluded as a candidate gene for ID due to its bi-allelic inheritance pattern, which is inconsistent with the autosomal dominant inheritance pattern observed in heterozygous CNVs.

Interestingly, four genes, namely *INTS13*, *PPFIBP1*, *REP15*, and *FAR2*, have emerged as strong candidates for KS coupled with ID at 12p11.2 (Fig. 2A).

*INTS13* (Integrator Complex Subunit 13, MIM 615079), also known as *ASUN* (Asunder, Spermatogenesis Regulator) is mapped 65 kb distal from the 12p11.23 telomeric breakpoint of the 4.7 Mb microdeletion. Although it is not directly encompassed in the 4.7 Mb deletion at 12p11.21-12p11.23, it might be dysregulated by a positional effect^29^ contributing to the KS phenotype seen in this Patient 1 with an unbalanced chromosome translocation^1^. *INTS13* plays a crucial role in spermatogenesis in *Drosophila melanogaster*, as demonstrated by studies showing spermatocyte arrest during prophase of meiosis I in Drosophila knockout models^98^. Additionally, germline expression of mouse Asun (Ints13) rescued sterility and dynein mislocalization in Asun mutant flies^99^. Three variants in this gene are reported in NDD patients (Table 2).

*PPFIBP1* (PPFIA Binding Protein 1, MIM 603141) is known to interact with TACR3 (Tachykinin Receptor 3, MIM 162332)^55^, a gene mutated in patients with hypogonadotropic hypogonadism^100^. Among the interacting proteins of PPFIBP1, YWHAG (Tyrosine 3-Monooxygenase/Tryptophan 5-Monooxygenase Activation Protein, Gamma Isoform, MIM 605356)^55^, KRAS (KRAS Protooncogene, GTPase, MIM 190070)^60^, NRAS (NRAS Protooncogene, GTPase, MIM 164790) and HRAS (HRAS Protooncogene, GTPase, MIM 190020)^101^, CUL3^102^, and SNAP29 (Synaptosomal-Associated Protein, 29-KD, MIM 604202)^55^ suggest a potential neurodevelopmental role for *PPFIBP1*. Notably, five missense variants in *YWHAG* were reported in patients with developmental and epileptic encephalopathy^103^, and *KRAS*^104^ and *NRAS*^105^ are associated with Noonan syndrome, whereas *HRAS* is involved in Costello syndrome^106^. Both syndromes share a neurodevelopmental phenotype along with distinct facial dysmorphism. Additionally, *CUL3* mutations have been associated with NDD^77^ and ASD^50^, While *SNAP29* is genetically associated with Cednik syndrome including neuropathy^107^, and schizophrenia^108^.

The second candidate gene, *REP15* (RAB15 Effector Protein, MIM 610848) interacts with SLC4A2 (Solute Carrier Family 4, Member 2, MIM 109280)^55^. Histopathologic analysis of *Slc4a2* KO mice revealed an interruption in spermiogenesis leading to infertility^109^. Moreover, REP15 effectively interacts with TLK2 (Tousled-Like Kinase 2, MIM 608439)^55^, which is associated with neurodevelopmental delay^110^, ASD^58^ and schizophrenia^111^.

*FAR2* (Fatty Acyl-CoA Reductase 2, MIM 616156), the third candidate gene for KS coupled with ID, physically interacts with the zona pellucida glycoprotein* 2* (*ZP2*, MIM 182888)^55^, variants of which were found in females with infertility^112–116^. FAR2 also interacts with ATP2B2 (ATPase, Ca (2+)-Transporting, Plasma membrane, 2, MIM 108733)^55,59^, variants of which are found in patients with ASD^58,117^. Additionally, FAR2 interacting with KCNA2 (Potassium Channel, Voltage-Gated, Shaker-Related Subfamily, Member 2, MIM 176262)^55^ is associated with epileptic encephalopathy^118^ and epilepsy^119–122^. Moreover, CUL3, another interacting protein of FAR2, is associated with NDDs^77^ and ASD^50^.

In conclusion, *INTS13*, *PPFIBP1, REP15,* and *FAR2* are strong candidate genes for KS in combination with NDDs at the 12p11.22-12p11.23 region.

*PTHLH* (parathyroid hormone like hormone MIM 168470) is associated with brachydactyly^123^ and explains this phenotype in the patient DCP308811. Conversely, bi-allelic variants of *IPO8* (importin 8 MIM 605600) have been linked to cardiovascular defects, skeletal anomalies, and immune dysregulation^124^. However, we excluded both genes from our study as their phenotypes were unrelated to KS and ID.

We propose that, despite not being encompassed by small CNVs we used in comparative genomic mapping, the expression levels of the two ID candidate genes *DENND5B*, and *ETFBKMT* could be altered due to position effect^125,126^. This likely explains the observed NDD phenotypes, including dystonia, global DD, growth delay, motor delay, etc., in one DECIPHER proband DCP288321 (Fig. 2A and Table 3).

To substantiate their pathogenicity, we also confirmed the high expression of our candidate genes in five different human tissues (i.e. brain, fetal brain, muscle, ovary, and testis) relevant to the phenotype of KS and NDD (Fig. 4B).

During the preparation of this manuscript, genome sequencing was performed on this same Patient 1 to map the translocation breakpoint. The analysis indicated that the deletion of *RMST* could potentially be a cause of KS due to loss of function, although this conclusion was based on an erroneous assumption that this chromosome translocation is balanced^12^. RMST physically interacts with SOX2^127^, a transcription factor known to regulate neural fate, and aids in the binding of SOX2 to the promoter of target genes important in neurogenesis^127^. Notably, *SOX2* (SRY-box transcription factor 2, MIM 184429) is a known disease gene for hypogonadotropic hypogonadism and combined pituitary hormone deficiency^128^. Additionally, *RMST* has been associated with rhabdomyosarcoma and melanoma^129^.

However, the ostensible pathogenicity of *RMST* in KS remains to be confirmed, as this Patient 1 has an unbalanced translocation accompanied by an additional 4.7 Mb microdeletion that we identified. Moreover, no mutations of this gene were found in the 48 KS patients we recruited. This case underscores the necessity and significance of aCGH or sequencing analysis in individuals with disease-associated, apparently balanced translocations to rule out cryptic microdeletions. Simultaneously, our study highlights the benefits of the integrated usage of karyotype analysis, aCGH, and sequencing for a comprehensive approach to phenotypic assessment.

In summary, our study revealed that an apparently balanced translocation t(7;12)(q22;q24)^1,11,12^ in Patient 1 is actually unbalanced, and the 4. 7 Mb cryptic deletion at 12p11.21-12p11.23 likely explains the phenotype of KS and ID in the patient carrying these two unrelated chromosomal rearrangements. Through in silico comparative genomic mapping with 14 additional CNVs in this genomic region, we identified one potential KS candidate gene (*TSPAN11*), seven candidate genes for NDD (*TM7SF3*, *STK38L, ARNTL2*, *ERGIC2*, *TMTC1*, *DENND5B,* and *ETFBKMT*), and four candidate genes for KS with ID (*INTS13, REP15*, *PPFIBP1*, and *FAR2*). The candidacy of these genes was further supported by their high-level expression pattern in the relevant human tissues. We propose that some dosage-sensitive genes in this genomic region might contribute to sexual and/or cognitive impairment in patients with KS, ID, or both, as indicated by the probabilities of dosage sensitivity (STK38L: pHaplo/pTriplo 0.77/0.94, DENND5B: 0.96/0.96, PPFIBP1: 0.81/0.66)^35^. This suggests that both increased or decreased expression can result in related deleterious phenotypes. To generate supporting evidence for this hypothesis, performing RT-qPCR or western blot analyses of candidate genes that are present in both the deletion and duplication regions will be crucial.

Considering the well-known phenomenon of heterogeneous neurodevelopmental phenotypes caused by mutations in the same gene^130^, our candidate genes at 12p11.21-12p11.23 present an opportunity to identify NDD disease genes from NGS databases containing a myriad of autosomal dominant or de novo variants of uncertain significance (VUSs).

## Patients and methods

### Human patients

This study was approved by the Augusta University Institutional Review Board (IRB) and was conducted following the principles outlined in the American Society of Human Genetics Code of Ethics. Participants were recruited by endocrinologists, gynecologists, or clinical geneticists for clinical characterization and genetic studies. Informed consent, approved by the Augusta University IRB, was obtained from all participants before conducting the genetic studies. The cohort comprised 48 American probands with KS or IHH and other accompanying minor phenotypes (24 women, 24 men). We isolated DNA from each participant’s peripheral blood, and subsequent mutation screening of positional candidate genes identified at both genomic breakpoints was performed. Notably, all 48 individuals tested negative for *ANOS1* and *FGFR1* variants. The KS diagnosis was based on the presence of IHH, which is characterized by delayed or absent pubertal maturation, along with low serum gonadotropins and sex steroids. Additionally, a smell deficit was identified from the patient's medical history and/or formal smell testing.

### Clinical reports

#### Patient 1- 4.7 Mb del(12)(p11.21p11.23), t(7;12)(q21.13;q23.1)*dn*

A detailed description of patient DGAP032, who presented with a balanced de novo reciprocal translocation, 46,XY,t(7;12)(q22;q24)*dn,* was previously published in 1990^1^. In summary, this 44-year-old Chippewa/French man exhibited hypogonadotropic hypogonadism, based upon low levels of FSH, LH, and testosterone, along with sparse pubic hair, small testes (<1 cm), olfactory deficiency, skeletal and cranial anomalies, and ID. He sought medical attention at the age of 22 years due to delayed sexual development. During the evaluation, normal 17-hydroxycorticosteroids and abnormally low 17-ketosteroids and gonadotropin levels were observed. The epiphyseal centers of most long bones and the spine were not yet closed, indicating delayed bone maturation. The bone age of the hand was estimated to be around 12 years, and the metacarpals appeared shortened and clubbed distal ends, especially the 4^th^ right metacarpal, indicative of brachydactyly. In addition, a sharply outlined foramen near the internal occipital protuberance of the occipital bone was noted. In 1984, lymphocyte chromosome studies demonstrated an apparently reciprocal translocation, t(7;12)(q22;q24), but upon further molecular analysis, it was revised as t(7;12)(q21.13;q23.1)*dn* (Fig. 1A)^11^. Clinical signs of KS were not observed in his five full sisters, one full brother, two half-brothers, or one half-sister.

#### Patient 2- 500 kb dup(12)(p11.23)

**Figure 5:**
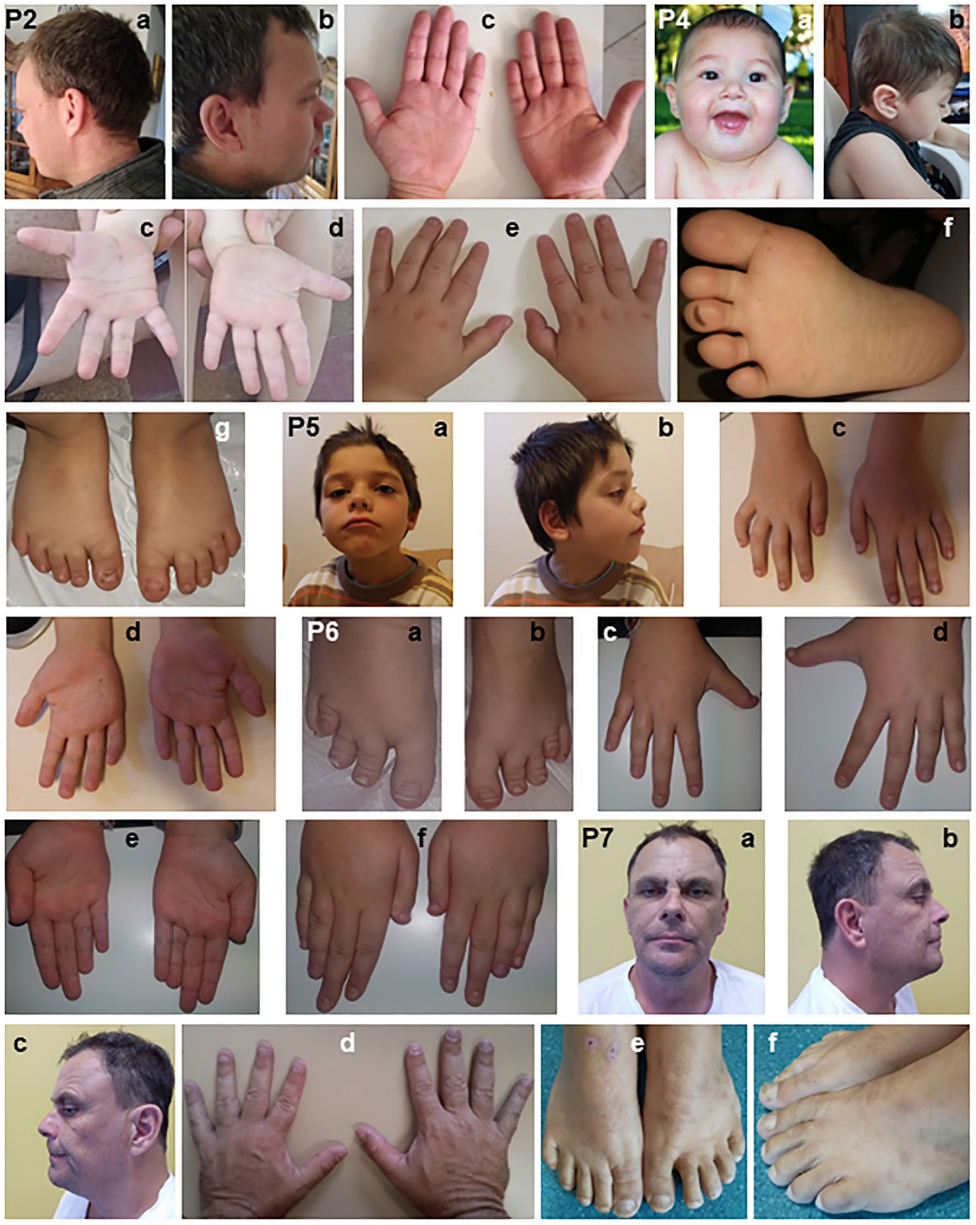
Facial and limb pictures of individuals with CNVs at 12p11.21-12p11.23. P2: Patient 2 on Table 1 (**a**) hypertelorism and slightly thicker lower lip (**b**) borderline posteriorly rotated right ear, (**c**) palms showing special form and disrupted vertical creases. P4: Patient 4 on Table 1 shows (**a**) microcephaly at 1 year (**b**) lateral view of right face at 3 years 10 months (**c**) hypoplasia of the vertical palmar flexion creases (PFC) on the right palm (**d**) the vertical PFC looks short and the transverse proximal looks with some tendency to Sydney line on the left palm (**e**) tapering fingers (**f**) a minor syndactyly between toes 2 and 3 (**g**) small toes and a questionable gap between toes 1 and 2. P5: Patient 5 on Table 1 shows (**a**) small forehead, right eyelid ptosis, bilateral downward palpebral fissures, frontal hair spike, lower lip eversion (**c**) tapering fingers and bilateral short 5th fingers with clinodactyly (**d**) the transverse proximal PFC has tendency to join the transverse distal, a variant of the PFC. P6: Patient 6 on Table 1 shows (**a**,**b**) short 4th toes likely due to short 4th metatarsals on both feet (**c**,**d**) dorsal view of tapering fingers with short 4th and 5th fingers in both hands (**e**) the transverse distal palmar flexion crease is rather short and doesn’t start at the ulnar margin on both palms (**f**) dorsal view of both hands showing bilateral short 4th fingers and short 5th fingers especially at right. P7: Patient 7 on Table 1 shows (**a**) essentially no dysmorphism (**b**,**c**) microcephaly (**d**) tapering fingers (**e**,**f**) clinodactyly of the 5th toes and a gap between toes 1 and 2 in the left foot.

Patient 2 (50943) is a 36-year-old man with a medical history including ID, DD, autism, and dyslexia. He was born with a birth weight of 3500 g. At 48 months of age, his IQ was assessed to be between 30 and 50. He achieved certain milestones independently, sitting at around 11 months, walking at 27 months, becoming toilet-trained by 2 years, and uttering his first words at 30 months. However, he also faced challenges such as learning disability, language delay, and speech delay. As he grew older, at 29 years of age, he was noted to frequently speak loudly and demand things with incomprehensible associations. While his tantrums were primarily verbal and seldom physical, they could be intense. He was extremely restless with no tics or stuttering, often repetitively using words and parrots “yes”. He spoke very loudly. Although no apparent physical abnormalities were observed, hypertelorism and a slightly thicker lower lip were noted. He did not have seizures, bone anomalies, facial dysmorphism, or shortened fingers or toes (Fig. 5). However, a brain MRI displayed an arachnoid cyst. Further analysis using aCGH on genomic DNA revealed a de novo 500 kb duplication at 12p11.23, resulting in 46,XY,arr[hg 38] (chr12:27,134,884-27,634,952)x3*dn*.

#### Patient 3- 750 kb dup(12)(p11.22p11.23)

Patient 3 (31606) is a 10-year-old female with a history of DD, speech delay*,* expressive language delays, and attention deficit hyperactivity disorder (ADHD)*.* At 3 days old, she had chronic diarrhea and struggled with poor growth or weight gain. Over the course of her early life, she was hospitalized four times between 52 days and 5 months of age due to failure to thrive and chronic diarrhea. Despite thorough testing, no conclusive diagnosis was reached during an 18-day hospitalization. Her chronic dehydration resulted from her body’s inability to absorb essential nutrients effectively as fluids rapidly passed through her system. A second gastroenterology opinion was sought, but no further testing was conducted. During infancy, she exhibited aversion to textured foods, necessitating feeding therapy. She was also diagnosed with congenital sucrose-isomaltase deficiency (CSID). She managed the milestone of walking at the age of 15 months, but her inability to crawl led to the need for occupational therapy. Additionally, she required social/emotional therapy. Early childhood education and speech therapy were implemented, and she later attended a mainstream school at 4.5 years old. aCGH analysis revealed a 750 kb duplication at 12p11.22-p11.23, resulting in 46,XX,arr[hg 38](chr12:27,157,806-27,907,534)x3.

#### Patient 4- 1.94 Mb dup(12)(p11.22)

Patient 4 (022821) is a four-year-old white male with a medical history of learning disability, dyslexia, hypotonia, language delays, and delayed speech. Born at full-term, via normal spontaneous delivery, his developmental milestones were delayed, with crawling at 7 months, sitting alone at 9 months, and walking unassisted at 2 years of age. Due to lack of strength in his abdomen, he needed some time to be able to sit alone. He began taking his first steps at 26 months, and he has been receiving occupational therapy (OT) since the age of 30 months. At 15 months, a neurologist diagnosed him with microcephaly (OFC 43 cm, approximately -3SD for age), short stature, DD, and impaired motor skills. Additionally, he has syndactyly and tapering fingers. At age 2 years, visual evoked potential testing detected some visual asymmetry. Maternal CMV infection and other intrauterine infections were ruled out. Though attentive to his surroundings, he showed no attempt to communicate. At 3 years and 10 months, it was discovered that he had moderate hearing loss in the left ear and mild hearing loss in the right ear (Fig. 5). An EEG performed at age 23 months showed normal results. Chromosomal analysis was normal, but a SurePrint-Ga Human Genome Kit Agilent aCGH (4x180K) revealed a 1.94 Mb duplication, 46,XY,arr[hg 38](chr12:28,047,313-29,990,575)x3 in 12p11.22. The patient presents characteristics of ASD, such as walking on tiptoe, repetitive movements, and obsession with spinning objects.

#### Patient 5- 215 kb dup(12)(p11.23)

Patient 5 (DCP295472) is 12 years and 5-month-old Caucasian male diagnosed with ASD and learning disability. He was born at full-term by spontaneous vaginal delivery, with a weight of 3.810 kg (76th centile), length of 50 cm (38th centile), and head circumference of 34 cm (22nd centile). He suffered from repetitive ear infections in childhood and underwent a surgery for ear tubes. He began walking at 24 months, but with language delay associated with a global DD and impaired motor skills. At the age of 2, neurobehavioral concerns arose, including repetitive movements, stereotypy, difficulties regulating emotions, and limited facial expressions. He also developed sleeping disorders, waking up early at 3-4 am, which were treated with melatonin. Food selectivity was observed, and at 10 years of age, his weight was 30.8 kg (median) with a height of 135 cm (median) and head circumference of 53 cm (median). Right eyelid ptosis and dysmorphic features, such as bilateral downward palpebral fissures, frontal hair spike, lower lip eversion were noted. Although an ophthalmic surgery was scheduled, the patient never attended the anesthesiologist appointment. Additionally, he also presented with clinodactyly of the 5^th^ finger on his right hand (Fig. 5). He attended a regular schooling at Abbotsford Virtual School. As part of his treatment plan, he was prescribed methylphenidate and melatonin. The patient underwent a chromosome Fragile X analysis and aCGH as part of the diagnostic tests. The chromosomal analysis showed a paternally inherited 215 kb duplication, 46,XY,arr[hg 38] (chr12:27,400,730-27,615,518)x3 *pat* at 12p11.23 and 407 kb deletion at 2q13, arr[hg 38] (chr2: 108,684,076-109,090,916)x1 *pat*. The father also presented with anxiety disorder, similar to his affected son, but follow-up with the father was limited.

#### Patient 6-2.18 Mb del(12)(p11.21p11.22)

Patient 6 (DCP370033) is an 11-year-old Caucasian girl with a history of DD, speech delay, learning difficulties, and ADHD. She is the first child of a healthy and non-consanguineous couple. The pregnancy was uncomplicated, and she was born full term by spontaneous vaginal delivery with a birth weight of 3 kg (99th centile). She has two younger sisters, one of whom has a unilateral third finger brachydactyly, as reported by her mother. The patient sat independently at approximately 7 months, started walking at 22 months, and began speaking her first words late. At age 5 years, speech delay with articulation problems became evident. Psychological tests revealed a clear discrepancy between performance and verbal capacities. She exhibited behavioral challenges including temper tantrums and a short attention span. Currently in the 3rd grade, she has mild ID and learning difficulties, with a poor attention span and easy distractibility. She received a special education with adapted curricular. She was seen in a neuropediatric clinic for recurrent headaches, which improved after discontinuing methylphenidate treatment. She is also under care in an endocrinology clinic for obesity. She has normal stature, normal head circumference and no dysmorphic features. She presents with a large thumb, shortening of the IV and V metacarpals and metatarsals, and tapering fingers without clinodactyly or syndactyly. She has short toes and a short 4^th^ metatarsal bone (Fig. 5). Her father with the same microdeletion had some learning difficulties and has similar foot abnormalities. However, he completed the 12th grade, as did his brothers, who were not tested for aCGH. The father’s brothers despite having more severe learning difficulties, are capable of living independently. aCGH (CGX-HD 180K by PerkinElmer®) revealed a paternally inherited 2.18 Mb deletion-arr[hg38] 12p11.21p11.22(chr12:28,414,984-30,598,365)x1 *pat* and a 49 kb duplication with unknown inheritance at Xp22.33, arr[hg38] (chrX:1,259,698-1,308,697)x3.

#### Patient 7- 652 kb dup(12)(p11.22)

Patient 7 (DCP293962) is a 48-year-old Caucasian male with a history of DD, dyslexia and ID with poor academic performance. He was born full term by spontaneous vaginal delivery with an average birth weight. His neonatal period was unremarkable. He achieved normal gross and fine motor milestones and displayed average social interactions during childhood. In early childhood, he was diagnosed with dyslexia and has speech delay. He could not read and can barely write his name. He left school in grade 8 and has been on a disability pension since then because he had difficulties holding a job. Despite his challenges, he does not have any disruptive or aggressive behavior. At the age of 42, he married and had the following physical characteristics: a height of 179 cm, weight of 95 kg, and head circumference of 59.2 cm. On examination, he was dysmorphic with upturned nostrils and a high nasal bridge. He also displayed clinodactyly and tapering fingers (Fig. 5), but his general examination was unremarkable. Fragile X analysis and urine metabolic screening yielded normal results. The aCGH analysis revealed a paternally derived microduplication: arr[hg38] 12p11.22 dup (chr12:28,701,107-29,353,047)x3 *pat,* and a 229 kb deletion with unknown inheritance at 16p13.3, arr[hg38] (chr16:6,699,348-6,927,950)x1*.* The father also had mild learning problems with the same syndactyly and tapering fingers.

### Eight decipher CNV patients in Fig. 2A

Brief clinical information, genomic coordinates, and inheritance patterns of eight Decipher CNV patients used in silico comparative genomic mapping in Fig. 2A are described in Table 3.

### Fluorescence in situ hybridization analysis (FISH)

A lymphoblastoid cell line (GM10565) from Patient 1, designated DGAP032 in the Developmental Genome Anatomy Project, was obtained from the NIGMS Human Genetic Cell Repository at the Coriell Institute for Medical Research (www.coriell.org)^10^. The karyotype, 46,XY,t(7;12)(q22;q24)*dn*, was reconfirmed prior to refinement of the breakpoint by FISH. Assignment of chromosome breakpoint locations to chromosomal bands was determined by GTG-banding. To identify genes potentially disrupted in the patient, translocation breakpoints were mapped using FISH. Maps from the National Center for Biotechnology Information (http://www.ncbi.nlm.nih.gov/genome/guide/human/)^131^ and the Genome Bioinformatics Group at the University of California Santa Cruz (http://genome.ucsc.edu/)^132^. Guided selection of BAC clones for breakpoint mapping was done by FISH. BAC clones from the RP11 (Children’s Hospital of Oakland Research Institute) and the CIT pool D (Research Genetics) libraries corresponding to relative locations on the UCSC map from chromosomes 7 and 12 were used as FISH probes on metaphase chromosome spreads from an Epstein-Barr virus-transformed lymphoblast cell line generated from the patient’s peripheral blood. Metaphase spreads were prepared according to a standard cytogenetic protocol. Human BAC clones were obtained from the RP11 (Children’s Hospital of Oakland Research Institute) and the CIT pool D (Research Genetics) libraries. BAC DNA was purified by alkaline lysis and isopropanol precipitation. After purification, BAC DNA was directly labeled by nick-translation with either SpectrumOrange or SpectrumGreen labeled nucleotides (Vysis) and used in single- or two-color FISH experiments. Slides were counterstained with 4’,6’-diamidino-2-phenylindole hydrochloride (DAPI). Representative metaphase images were recorded using the CytoVision image analysis system (Applied Imaging) database.

Using the relative STS positions on the UCSC map, BAC clones were chosen to cross the relevant regions on chromosomes 7 and 12. FISH analysis of each clone was then used to identify clones that mapped proximal or distal to each chromosome breakpoint. By this way, physical maps of chromosomes 7q21 and 12q24 were constructed, and the breakpoint regions narrowed and defined^11^.

### Southern blot analysis

Southern blot analysis of patient lymphoblast genomic DNA with seven probes (from KS-1 to KS-7) to search for altered restriction fragments was carried out using standard protocols (Fig. 3B). For each lane, 10 µg of genomic DNA from the patient and control were digested with an appropriate restriction enzyme. Fragments were separated on a 1.0% agarose gel and transferred to Hybond-N membrane (Amersham, Arlington Heights, Illinois, USA). Filters were ultraviolet cross linked, baked at 80 °C, and hybridized with probes labelled with 32P-dCTP by random priming. Hybridization of labelled fragments was done in the presence of excess herring sperm competitor DNA, and hybridized membranes were washed at 60 °C with 0.15 M NaCl/0.015 M sodium citrate/0.1% sodium dodecyl sulphate (SDS) for 30 mins. Autoradiography took place for 16 hours at – 70 °C using two intensifying screens. Seven hybridization probes were amplified by the primer sets mentioned in Supplementary Table 1. After the breakpoint region was apparently narrowed to 3.5 kb between CTD-2268E11 and CTD-2542D2 at chromosome 12 in band q23 by FISH, the first four genomic probes, KS-1, KS-2, KS-3, and KS-4, within this region were amplified from the breakpoint spanning BAC clone RP11-492N15 under the following conditions (Figs. 1B, 2B and 3A):: initial denaturation at 94 °C for 2 min, followed by 30 cycles at 94 °C for 30 sec, 58 °C for 30 sec, 72 °C for 45 sec (KS-1, KS-2, and KS-3) or 3 min for 30 sec (KS-4), and extension at 72 °C for 5 min after the last cycle. Two genomic probes KS-5 and KS-6 were amplified from this region (Fig. 3A) using BAC RP11-492N15 under the following conditions: initial denaturation at 94 °C for 2 min, followed by 30 cycles at 94 °C for 30 sec, 63 °C for 30 sec, 72 °C for 50 sec, and extension at 72 °C for 5 min after the last cycle. The probe KS-7 was amplified within that putative breakpoint region using BAC RP11-492N15 under the following conditions: initial denaturation at 94 °C for 2 min, followed by 30 cycles at 94 °C for 30 sec, 58 °C 30 sec, 72 °C for 40 sec, and extension at 72 °C for 5 min after the last cycle. The list of primers used for the amplification of probes for Southern hybridization is presented in Supplementary Table 1.

### Suppression PCR and nested PCR

The 3.5 kb junction fragment from der(12) was amplified by suppression PCR using the following primer sets and the conditions. Primers flanking the 1.5 kb narrowed breakpoint region from the 12q23.1 were used with adaptor-based primers: PCR1): AP1-A 5′CCTAATACGACTCACTATAGG3′ + AC007351-54209rev 5′GTGAATGGTGGATAGTGCTC3′; AP2-A 5′CTATAGGGCTCGAGCGGC3′ + AC007351-54176rev 5′GATTAAATTCACTCTCTGAAGAA3′. PCR2): AP1-A 5′CCTAATACGACTCACTATAGG3′ + AC007351-54176rev 5′ GATTAAATTCACTCTCTGAAGAA3′; AP2-A 5′CTATAGGGCTCGAGCGGC3′ + AC007351-54026rev 5′CTAGCTTACAATTTTCTGGTGA3′. Initial denaturation was at 94 °C for 2 min, followed by 30 cycles at 94 °C for 30 sec, 57 °C for 30 sec, 72 °C for 1 min 30 sec, and extension at 72 °C for 5 min after the last cycle.

The 2.3 kb junction fragment from der(7) was amplified by nested PCR using the following primer sets and the conditions. Initial denaturation at 94 °C for 2 min, followed by 30 cycles at 94 °C for 30 sec, 57 °C for 30 sec, 72 °C for 2 min 30 sec, and extension at 72 °C for 5 min after the last cycle. PCR1)^11^: 5′CCATTGGCTTTAAGTGTATAGT3′+ 5′CTTGTGTGTACATCTCCTGAA3′; PCR2): 5′CAACAGACATCTGCATTTACTT3′+5′GAAGATAGCTATAACAACAGC3′.

### Mutation screening of five genes at the breakpoints of t(7;12)(q21.13;q23.1)*dn*

We screened for mutations in five genes - *RMST*, *NEDD1*, *PAFAH1B2P2*, *ZNF804B,* and *STEAP4 -* in 48 KS patients including our translocation patient, who was also screened for *FGFR1* (Fibroblast Growth Factor Receptor 1, MIM 136350) and *ANOS1* (Anosmin 1, MIM 300836) to exclude the possibility of mutations in these two well-known genes with high prevalence for KS. A combination of single strand conformation polymorphism analysis (SSCP) and direct sequencing of *RMST* and *ZNF804B* were performed in mutation screening. The 46 primer sets were designed to cover all exons and flanking intronic regions of two predicted mRNAs. The size of amplicon is adjusted to less than 350 bp for SSCP, and a few parts of mRNAs were applied on PCR and direct sequencing to check for a mutation. PCR was carried out with 10 ng of genomic DNA of the patient’s sample or normal control in 20 ul of reaction (primer sequences and amplification conditions are available on request). Then PCR products were electrophoresed on precast gels of ExcelGel DNA Analysis Kit (Amersham Biosciences) according to the manufacturer’s instructions. Sequentially, the DNA gel was stained by silver stain according to the manufacturer’s instructions (DNA silver staining kit; Amersham Biosciences) to visualize and permanently stain the discrete DNA bands. When aberrant band patterns were recognized on the samples compared with normal controls, PCR products that have the aberrant band on the gel were sequenced with ABI Prism 377 sequencer (Applied Biosystems, Foster City, Calif.). Sequences were aligned and compared with sequences of predicted mRNAs to confirm the mutation. For seven genes, all coding regions and exon-intron boundaries were directly amplified and sequenced. NCBI reference mRNA sequences used for screening were NR_152618.1 (*RMST*), NM_001135175.1 (*NEDD1*), NR_077240.1 (*PAFAH1B2P2*), NM_181646.5 (*ZNF804B*), NM_024636.4 (*STEAP4*), NM_001174067.1 (*FGFR1*), and NM_000216.4 (*ANOS1*).

### aCGH (array comparative genomic hybridization)

DNA extracted from the cell line was compared to a reference sample for standard two-color aCGH. Reference DNA was purchased from Promega (Madison, WI, USA). Test samples were labeled using Cy5 and reference DNA was labeled using Cy3. Agilent 244K human genome oligonucleotide aCGH (G4411B) was used for aCGH analysis following the manufacturer’s instructions (Oligonucleotide Array-Based CGH for Genomic DNA Analysis protocol version 3 (Agilent Technologies, Palo Alto, CA, USA). Images were captured using an Agilent scanner and quantified using Feature Extraction software v9.0 (Agilent Technologies, Palo Alto, CA, USA). CGH analytics software v3.4 (Agilent Technologies, Palo Alto, CA, USA) was subsequently used for data normalization, quality evaluation, and data visualization. Copy number aberration was indicated using the ADM-2 (Aberration Detection Method 2) algorithm. Probe positions were mapped to GRCh38.

### In silico comparative CNV mapping

The phenotypes from our seven CNV patients (Patients 1–7), DGAP032, 50943, 31606, 022821, 295472, 370033 and 293962 (Table 1) were compared with eight unpublished CNV cases from the DECIPHER database. Genomic coordinates from these cases were converted to hg38 before the comparison was carried out (Table 3). Three factors were considered for choosing candidate genes – (1) sporadic genetic variants reported in humans with matching phenotypes, (2) knockdown or knockout animal models recapitulated human phenotypes, (3) their interacting proteins were investigated, and the genetic variants of corresponding genes reported in human patients with a similar phenotype (Table 2). Literature was also reviewed as well as several databases including Human Gene Mutation Database HGMD Professional (2022.2) (https://my.qiagendigitalinsights.com/bbp/view/hgmd/pro/start.php), MGI (6.21) (Mouse Genome Informatics) (http://www.informatics.jax.org/), BioGrid (4.4.212) (Database of Protein, Chemical, and Genetic Interactions, thebiogrid.org), and VarElect (https://varelect.genecards.org/).

### Quantitative reverse transcription PCR (RT-qPCR)

RT-qPCR was performed from total RNA of five different human tissues including brain, fetal brain, muscle, ovary and testis. Catalog numbers of the five tissues obtained from Clontech were as follows: brain- 636530, fetal brain total-636526, muscle- 636534, ovary- 636555 and testis- 636533. cDNA synthesis was performed using 1-2 μg of total RNA using high-Capacity cDNA Reverse Transcription Kit and analyzed by RT-PCR on QuantStudio 6 Flex system using SYBR Green (ThermoFisher, Waltham, MA). The ΔCt method was used to calculate the relative expression of each gene. In conclusion, the difference between the Ct values (Ct) of the target gene and the reference gene, *GAPDH*, was used to compute relative gene expression. After determining ΔCt, the fold change (2−ΔCt) was calculated, and the relative expression was plotted as excel graphs.

### Ethics declarations

The studies involving human participants were reviewed and approved by the Institutional Review Board of Augusta University, Georgia, USA.

### Consent to publish

Written informed consent for publication of any potentially identifiable images or data included in this article was obtained from all study participants, and/or respective parent(s) and/or legal guardians.

### Supplementary Information

Supplementary Table 1.

## Data Availability

The genomic coordinates and phenotypes of seven patients with CNVs are listed in Table 1. Out of those, three CNVs of Subjects 2, 3, and 4, which were not previously reported, were submitted and are available at the Leiden Open Variation Database (https://www.lovd.nl/3.0/home) under the individual ID numbers 00433002, 00433003, and 00433004 with two links below. https://databases.lovd.nl/shared/individuals?search_Individual/Reference=Kim%202023 and https://databases.lovd.nl/shared/variants?search_VariantOnGenome/Reference=Kim%202023.

## Abbreviations

KS: Kallmann syndrome
CNV: Copy number variation
NDDs: Neurodevelopmental disorders
IHH: Idiopathic hypogonadotropic hypogonadism
ID: Intellectual disability
DD: Developmental delay
ADHD: Attention deficit hyperactivity disorder
aCGH: Array comparative genomic hybridization
CFA: Craniofacial anomalies
GnRH: Hypothalamic gonadotropin releasing hormone
BAC: Bacterial artificial chromosome
FISH: Fluorescence in situ hybridization
