# Supplementary material for: A cryptic microdeletion del(12)(p11.21p11.23) within an unbalanced translocation t(7;12)(q21.13;q23.1) implicates new candidate loci for intellectual disability and Kallmann syndrome
Source: Sci Rep. 2023 Aug 10;13:12984. doi: 10.1038/s41598-023-40037-4 (PMC10415337; doi:10.1038/s41598-023-40037-4)
Supplement: Supplementary file 1 — Supplementary Table 1. [file 41598_2023_40037_MOESM1_ESM.docx]

| **Probe name** | **Fragment size (bp)** | **Forward primer (5'-3')** | **Reverse primer (5'-3')** |
| --- | --- | --- | --- |
| **KS-1** | **525** | **CAGCGTGCTGAATATCACCA** | **TCAAGTCAGCAGTCTCTCTC** |
| **KS-2** | **618** | **CCTGGACCTTAGTAGATTACA** | **GTGCTGGGATTAGGACAAGA** |
| **KS-3** | **353** | **GAACATGTGTCTCTTTGTAAGG** | **TTCTGAAGACAACAGAAGTGG** |
| **KS-4** | **3341** | **CTCATCTATGTGTACACTTGC** | **TTCTGAAGACAACAGAAGTGG** |
| **KS-5** | **605** | **CATGAATCCTTGCCAACAAGG** | **GCTGAATTAACACACCATTAGTG** |
| **KS-6** | **392** | **CATAGCCTCTTAATCACCAAGG** | **CGGATGATGCAGCTCTAGGT** |
| **KS-7** | **497** | **GAGACTCCTAATTCTCCTAAGG** | **CTCCTCGGTATGTATCTATTATG** |

**Suppl. Table 1.** Primers used for amplification of PCR-derived probes applied for mapping of the 12q23 translocation breakpoint by Southern blot hybridization.
